# Dataset for characterization of thrombospondin family in chum salmon (*Oncorhynchus keta*)

**DOI:** 10.1016/j.dib.2019.01.008

**Published:** 2019-01-09

**Authors:** Sang Yoon Lee, Yi Kyung Kim

**Affiliations:** Gangneung-Wonju National University, Republic of Korea

## Abstract

An extracellular matrix (ECM) is composed of multiprotein networks for stable interactions between cells. Thrombospondins (TSPs) are known to exert extracellular matrix interactions, synapse formation, angiogenesis and immune response in vertebrates. A five TSP gene family is divided into two subfamilies on basis of their domain architecture. Until recently, exploitation of diverse TSP genes in teleost has been still limitedly exemplified. Therefore, we report the cDNA structures and expression profiles for TSPs-1, 2, 3A, 3B, and 4B of chum salmon, *Oncorhynchus keta*. In conjunctional with bioinformatics analysis, the diverse domain structures of TSP genes are identified. In addition, the major domain, repeat and motif of TSP isoforms of chum salmon were aligned with those of other salmonid fishes.

**Specifications table**

**Table:** 

Subject area	Biology
More specific subject area	Molecular biology
Type of data	Table, graph, sequence
How data were acquired	Quantitative RT-PCR, 3730xl DNA analyzer
Data format	Analyzed
Experimental factors	Smolt stage of chum salmon growth developmental stage
Experimental features	Tissue distribution of chum salmon thrombospondin cDNA genes by RT-PCR analysis in various tissues
	cDNA Cloning and characterization of chum salmon thrombospondin family
	Analysis of chum salmon thrombospondin family amino acid sequence domains
Data source location	Korea Fisheries Resources Agency (FIRA), Yangyang-gun, South Korea
Data accessibility	The thrombospondin family cDNA sequences are registered in the NCBI with accession number MK139486MK139490
Related research article	Duplication and distinct expression patterns of two thrombospondin-1 isoforms in teleost fishes [1]

**Value of the data**

• This is the first data to investigate the tissue expression profiles of teleost TSP-1, 2, 3A, 3B and 4B isoforms and may be used as a basis for TSP isoforms researches of other vertebrates, including mammals, birds, amphibians and teleost fishes [1].
• Based on the tissue distribution data of TSP isoforms, it may be useful to select major tissues involved angiogenesis, apoptosis, the activation of transforming growth factor β and immune regulation in teleost species.
• Various domains present in TSP have been reported to interact with extracellular matrix or cell surface receptors and various sequences of molecules. Therefore, the present data can contribute to the TSP function of salmonid fishes based on the relationship between the types and distribution information of various domains present in chum salmon TSP isoforms and expression characteristics.

## 1 Data

**Fig. 1:**
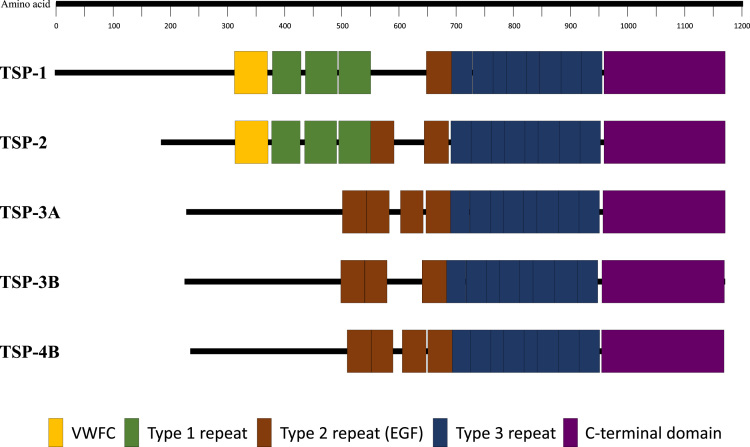
Schematic representation of chum salmon TSP family of proteins.

**Fig. 2:**
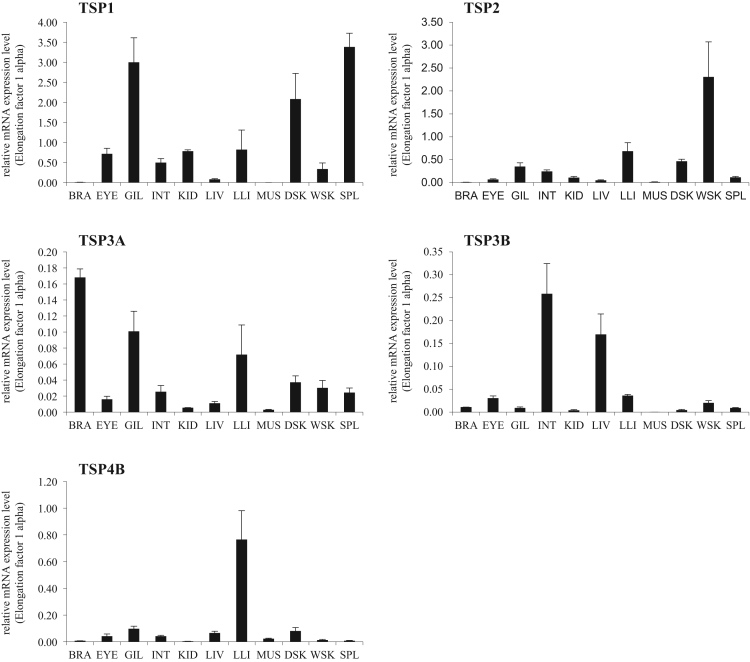
Expression of chum salmon TSP isoform gene transcripts detected by real-time RT-PCR. BRA: brain; GIL: gill; INT: intestine; KID: kidney; LIV: liver; MUS: muscle; DSK: dark skin; WSK: white skin; SPL: spleen.

The schematic diagram of domain architectures of each isoform of chum salmon are shown in Fig. 1. In order to compare TSPs structure between salmonid fishes, multiple alignments were performed for each domain or cDNA ORF sequences in TSP isoforms (Table 2 and Supplementary A and B). In addition, the tissue distributions of chum salmon TSP isoforms were investigated by real-time quantitative RT-PCR (Fig. 2). Identification of the specific amplifications for each isoform was confirmed by 1.5% agarose gel electrophoresis of the PCR products and melting curve analysis (Supplementary C).

## 2 Experimental design, materials, and methods

### 2.1 Fish and sample collection

The chum salmon fry, one month after hatching, was transferred from FIRA to the laboratory. Experiments were conducted with smolt-stage chum salmon after laboratory-reared 6 months. Chum salmon was raised at water temperature of 18±1 °C and dissolved oxygen content of 8.0 ± 0.5 mg/L. The average weight and total length were about 26 g and 15.7 cm, respectively. Before the procedure of dissection, the fish were anesthetized by 2-phenoxyethanol (Sigma Aldrich Co, St. Louis, USA) anesthesia and tissue samples were extracted. All tissue samples were immediately stored in a −80 °C cryogenic freezer.

### 2.2 Total RNA isolation, cDNA synthesis and cloning of cDNA

Total RNA was extracted from chum salmon tissues with RNAiso Reagent (Takara Bio, Shiga, Japan), according to the manufacturer׳s instructions. In addition, total RNA was repurified using RNeasy Plus Mini Kit (Qiagen, Hilden, Germany) and RNase-free DNase set (Qiagen) according to the manual. The total RNA (0.5 µg) from each sample was synthesized to cDNA using the PrimScript RT reagent Kit (Takara) with random primer and oligo-dT. Degenerate primers were designed in a conserved region based on the TSP isoforms cDNA sequence of salmonid species. And the full-length cDNAs of TSP isoforms were obtained by the rapid amplification of the cDNA ends (RACE) method using a FirstChoiceTM RLM-RACE Kit (Invitrogen, Carlsbad, USA).

### 2.3 Bioinformatics analysis

The completed sequence of open reading frame (ORF) of each TSP isoform was found using ORF Finder (https://www.ncbi.nlm.nih.gov/orffinder/). Homologous sequences of TSP-1, TSP2, TSP3A, TSP3B and TSP4B genes were predicted in GenBank with the BLASTx (https://blast.ncbi.nlm.nih.gov/Blast.cgi). Characteristic domains, repeats, motifs and conserved cysteine residues forming the disulfide bonds in the protein sequence were predicted by ExPASy PROSITE (http://prosite.expasy.org/). Multiple alignments of the protein sequences were analyzed using ClustalW (http://www.genome.jp/tools-bin/clustalw).

### 2.4 Real-time PCR and statistical analysis

Real time quantitative RT-PCR was performed to detect mRNA expression levels of TSP isoforms in various tissues using a Thermal Cycler DiceTM real-time PCR system (Takara). The gene-specific primer pairs for RT-PCR were designed based on the cloned cDNA sequences of TSP isoforms (Table 1). The amplification efficiencies with primer designed for PCR assays ranged between 1.9 and 2.0. The elongation factor 1-alpha (EF1a) gene was used as internal control gene [2]. The real time RT-PCR reaction was used SYBR premix Ex TaqII Kit (Takara). The real time RT-PCR was performed in triplicate according to the following conditions: 95 °C for 30 s, followed by 45 cycles at 95 °C for 5 s and 60 °C for 30 s respectively. The melting curve analysis was carried out at the end of each real time RT-PCR run by increasing the temperature in a stepwise manner by 0.5 °C every 5 s, from 60 °C to 95 °C.

**Table 1:** Nucleotide sequences of quantitative real-time RT-PCR primers.

**Primer name**	**Gene**	**Sequences (5′-3′)**
qOK_TSP1_FW1	Thrombospondin 1	GACTTCACAGCCTACAGATG
qOK_TSP1_RV1		CCATCTCTTGCGAGAAGACA
qOK_TSP2_FW1	Thrombospondin 2	AGGATTACACAGCCTACAGG
qOK_TSP2_RV1		GAGAAGAAGACCAGTTCCTG
qOK_TSP3A_FW1	Thrombospondin 3A	TGGCGTGGTGATTGACACTA
qOK_TSP3A_RV1		CTTGATGTGCAGCAGAACCT
qOK_TSP3B_FW1	Thrombospondin 3B	ATGACATTGGTGGCAGACTC
qOK_TSP3B_RV1		GATCTGCTTGCGGTAGAACT
qOK_TSP4B_FW1	Thrombospondin 4B	GCAGTGAAGTCTAAGACAGG
qOK_TSP4B_RV1		AACCTGTGGTCTGTGCTGTA
qOK_EF1A_FW1	Elongation factor 1-alpha	AGCTCAAGGAGAAGATCGAC
qOK_EF1A_RV1		CTTGATGACACCAACAGCCA

**Table 2:** The basic information of chum salmon TSP isoform genes.

**Gene**	**Accession no.**	**ORF length (bp)**	**Size (AA)**	**Mw (kD)**	**pI**
Thrombospondin 1	MK139486	3519	1172	130.098	4.70
Thrombospondin 2	MK139487	2961	986	108.992	4.53
Thrombospondin 3A	MK139488	2868	955	104.846	4.40
Thrombospondin 3B	MK139489	2868	955	104.453	4.52
Thrombospondin 4B	MK139490	2844	947	103.655	4.38

All data from the qPCRs were expressed as mean ± standard error of the mean (SEM) and carried out by one-way ANOVA with a significance level of *p* < 0.05 using SPSS 25.0 software.

## Appendix A Supplementary material

Supplementary material **Supplementary A.** Multiple alignments of each domain, repeat and motif in TSP isoforms from salmonid fishes. OKE: *Oncorhynchus keta*; OKI: *Oncorhynchus kisutch*; OTS: *Oncorhynchus tshawytscha*; OMY: *Oncorhynchus mykiss*; SSA: *Salmo salar*; SAL*: Salvelinus alpinus*.

.

Supplementary material **Supplementary B**. Multiple alignment of chum salmon TSP cDNA isoforms.

.

Supplementary material **Supplementary C**. Agarose gel electrophoresis of RT-PCR products show single amplicons (A) and melt curves from qPCR of chum salmon TSP isoform and EF1a genes (B).
