# Supplementary material for: Dataset for characterization of thrombospondin family in chum salmon (Oncorhynchus keta)
Source: Data Brief. 2019 Jan 9;22:866–70. doi: 10.1016/j.dib.2019.01.008 (PMC6362859; doi:10.1016/j.dib.2019.01.008)
Supplement: Supplementary file 1 — Supplementary material [file mmc1.pdf]

Author’s declaration

We declare that there is no conflict of interest with regard to this publication.

We confirm that the manuscript has been approved for read and written by all authors.

Sign this document

- Full name & E-mail: Sang Yoon Lee (lsy0423@gwnu.ac.kr)

- Signature 2018. 11. 19

- Full name & E-mail: Yi Kyung Kim (yikyung1118@gwnu.ac.kr)

- Signature 2018. 11. 19
