# Supplementary material for: Dataset for characterization of thrombospondin family in chum salmon (Oncorhynchus keta)
Source: Data Brief. 2019 Jan 9;22:866–70. doi: 10.1016/j.dib.2019.01.008 (PMC6362859; doi:10.1016/j.dib.2019.01.008)
Supplement: Supplementary file 2 — Supplementary material Supplementary A. Multiple alignments of each domain, repeat and motif in TSP isoforms from salmonid fishes. OKE: Oncorhynchus keta; OKI: Oncorhynchus kisutch; OTS: Oncorhynchus tshawytscha; OMY: Oncorhynchus mykiss; SSA: Salmo salar; SAL: Salvelinus alpinus. [file mmc2.pdf]

OK_TSP1

VWFC (residues 317-374)

```
OTS   GVCLHNGIVHKNKAEWTVDDCTECTCQNSATVCRKISCPLIPCANATVPDGECCPRCG
OKE   GVCLHNGIVHKNKAEWTVDDCTECTCQNSATVCRKISCPLIPCANATVPDGECCPRCG
OMY   GVCLHNGIVHKNKAEWTVDDCTECTCQNSATVCRKISCPLIPCANATVPDGECCPRCG
SSA   GVCLHNGIVHKNKAEWTVDDCTECTCQNSATVCRKISCPLIPCANATVPDGECCPRCG
SAL   GVCLHNGIVHKNKAEWTVDDCTECTCQNSATVCRKISCPLIPCANATVPDGECCPRCG
      **********************************************************
```

EGF (residues 648-692)

```
OTS   PRNPCLDGSHDCNKNARCNYLGQFADPMYRCECKPGYAGNGHICG
OKE   PRNPCLDGSHECNKNARCNYLGQFADPMYRCECKPGYAGNGHICG
OMY   PRNPCLDGSHDCNKNARCNYLGQFTDPMYRCECKPGYAGNGHICG
SSA   PRNPCLDGSHDCNKNARCNYLGHFADPMYRCECKPGYAGNGHICG
SAL   PRNPCLDGSHDCNKNARCNYLGHFADLMYRCECKPGYAGNGHICG
      **********:***********:*:* ******************
```

Type 1 (residues 382-431)

```
OTS   DGWSLWSEWTHCSVSCGRGIQQRGRSCDRINNNCEGTSVQTRDCYLQECD
OKE   DGWSLWSEWTHCSVSCGRGIQQRGRSCDRINNNCEGTSVQTRDCYLQECD
OMY   DGWSLWSEWTHCSVSCGRGIQQRGRSCDRINNNCEGTSVQTRDCYLQECD
SSA   DGWSLWSEWTHCSVSCGRGIQQRGRSCDRINNNCEGTSVQTRDCYLQECD
SAL   DGWSLWSEWTHCSVSCGRGIQQRGRSCDRINNNCEGTSVQTRDCYLQECD
      **************************************************
```

Type 1 (residues 437-492)

```
OTS   DGAWSHWSPWSSCSVTCGAGVITRIRLCNSPTPQLEGKDCQGEGRQTETCTKSPCP
OKE   DGAWSHWSPWSSCSVTCGAGVITRIRLCNSPTPQLGGKDCQGEGRQTETCTKSPCP
OMY   DGAWSHWSPWSSCSVTCGAGVITRIRLCNSPTPQLEGKDCQGEGRQTEKCTKSPCP
SSA   DGAWSHWSPWSSCSVTCGAGVITRIRLCNSPTPQLEGKDCQGEGRQTEKCTKSPCP
SAL   DGAWSHWSPWSSCSVTCGAGVITRIRLCNSPTPQLGGKDCQGEGRQTEKCTKSPCP
      *********************************** ************.*******
```

Type 1 (residues 494-549)

```
OTS   NGNWGPWSLWDTCSATCGGGAQTRKRLCNDPAPKYGGKECQGDSKATQQCNKNACP
OKE   NGNWGPWSLWDTCSATCGGGAQTRKRLCNDPAPKYGGKECQGDSKATQQCNKNACP
OMY   NGNWGPWSLWDTCSATCGGGAQTRKRLCNDPAPKYGGKECQGDSKATQQCNKNACP
SSA   NGNWGPWSLWDTCSATCGGGAQTRKRLCNDPAPKYGGKECQGDPKATQLCNKNTCP
SAL   NGNWGPWSLWDTCSATCGGGAQTRKRLCNDPXPKYGGKECQGDSKASQQCNKNACP
      ******************************* ***********.**:* ****:**
```

Type 3 (residues 693-728)

```
OTS   EDTDLDGWPNQDLVCVENATYHCKKDNCPNLPNSGQ
OKE   EDTDLDGWPNQDLVCVENATYHCKKDNCPNLPNSGQ
OMY   EDTDLDGWPNQDLVCVENATYHCKKDNCPNLPNSGQ
SSA   EDTDLDGWPNQDLVCVENATYHCKKDNCPNLPNSGQ
SAL   EDTDLDGWPNQDLVCVENATYHCKKDNCPNLPNSGQ
      ************************************
```

Type 3 (residues 729-764)

```
OTS   EDYDKDGVGDACDNDDDNDGIPDDRDNCPFVYNPRQ
OKE   EDYDKDGVGDACDNDDDNDGIADDRDNCPFVYNPRQ
OMY   EDYDKDGVGDACDNDDDNDGIADDRDNCPFVYNPRQ
SSA   EDYDKDGVGDACDNDDDNDGIADDRDNCPFVYNPRQ
SAL   EDYDKDGVGDACDNDDDNDGIADDRDNCPFVYNPRQ
      *********************.**************
```

Type 3 (residues 765-787)

```
OTS   YDYDRDDIGDRCDNCPYNSNPDQ
OKE   YDYDRDDIGDRCDNCPYNSNPDQ
OMY   YDYDRDDIGDRCDNCPYNSNPDQ
SSA   YDYDRDDIGDRCDNCPYNSNPDQ
SAL   YDYDRDDVGDLCDNCPYNSNPDQ
      *******:** ************
```

Type 3 (residues 788-823)

```
OTS   TDTDNNGEGDACSVDIDGDGILNEKDNCPYVYNVDQ
OKE   TDTDNNGEGDACSVDIDGDGILNEKDNCPYVYNVDQ
OMY   TDTDNNGEGDACSVDIDGDGILNEKDNCPYVYNVDQ
SSA   TDTDNNGEGDACSVDIDGDGILNEKDNCPYVYNVDQ
SAL   TDTDNNGEGDACSVDIDGDGILNEKDNCPYVYNVDQ
      ************************************
```

Type 3 (residues 824-846)

```
OTS   KDTDLDGVGDMCDNCPLEHNPDQ
OKE   KDTDLDGVGDMCDNCPLEHNPDQ
OMY   RDTDLDGVGDMCDNCPLEHNPDQ
SSA   RDTDLDGVGDMCDNCPLEHNPDQ
SAL   RDTDLDGVGDMCDNCPLEHNPDQ
      :**********************
```

Type 3 (residues 847-884)

```
OTS   VDSDDDRVGDKCDSNQDIDEDGHQNNLDNCPYIPNANQ
OKE   VDSDDDRVGDKCDSNQDIDEDGHQNNLDNCPYIPNANQ
OMY   VDSDDDRVGDKCDSNQDIDEDGHQNNLDNCPYIPNANQ
SSA   VDSDDDRVGDKCDSNQDIDEDGHQNNLDNCPYIPNANQ
SAL   VDSDDDRVGDKCDSNQDIDEDGHQNNLDNCPYIPNANQ
      **************************************
```

Type 3 (residues 885-920)

```
OTS   ADHDKDGKGDACDHDDDNDGIPDEKDNCRLAFNPDQ
OKE   ADHDKDGKGDACDHDDDNDGIPDEKDNCRLAFNPDQ
OMY   ADHDKDGKGDACDHDDDNDGIPDEKDNCRLAFNPDQ
SSA   ADHDKDGKGDACDHDDDNDGIPDEKDNCRLAFNPDQ
SAL   ADHDKDGKGDACDHDDDNDGIPDEKDNCRLAFNPDQ
      ************************************
```

Type 3 (residues 921-956)

```
OTS   IDSDGDGRGDACKDDFDQDNVPDIYDVCPENFDISE
OKE   IDSDGDGRGDACKDDFDQDNVPDIYDVCPENFDISE
OMY   IDSDGDGRGDACKDDFDQDNVPDIYDVCPENFDISE
SSA   IDSDGDGRGDACKDDFDQDNVPDIYDVCPENFDISE
SAL   IDSDGDGRGDACKDDFDQDNVPDIYDVCPENFDISE
      ************************************
```

TSP-CTER (residues 960-1172)

```
OTS   RKFQMVPLDPKGTSQIDPNWVVRHQGKELVQTVNCDPGIAVGFDEFNSVDFSGTFFINTERDDDYAGFVFGYQSSSRFYVVMWKQITQTYWNNKPTKAQGYSGLSIKVVNSTTGPGEHLRNALWHTGNTPGQVKTLWHDPKNIGWKDF
OKE   RKFQMVPLDPKGTSQIDPNWVVRHQGKELVQTVNCDPGIAVGFDEFNSVDFSGTFFINTERDDDYAGFVFGYQSSSRFYVVMWKQITQTYWNNKPTKAQGYSGLSIKVVNSTTGPGEHLRNALWHTGNTPGQVKTLWHDPKNIGWKDF
OMY   RKFQMVPLDPKGTSQIDPNWVVRHQGKELVQTVNCDPGIAVGFDEFNSVDFSGTFFINTERDDDYAGFVFGYQSSSRFYVVMWKQITQTYWNNKPTKAQGYSGLSIKVVNSTTGPGEHLRNALWHTGNTPGQVKTLWHDPKNIGWKDF
SSA   RKFQMVPLDPKGTSQIDPNWVVRHQGKELVQTVNCDPGIAVGFDEFNSVDFSGTFFINTERDDDYAGFVFGYQSSSRFYVVMWKQITQTYWNNKPTKAQGYSGLSIKVVNSTTGPGEHLRNALWHTGNTPGQVKTLWHDPKKIGWKDF
SAL   RKFQMVPLDPKGTSQIDPNWVVRHQGKELVQTVNCDPGIAVGFDEFNSVDFSGTFFINTERDDDYAGFVFGYQSSSRFYVVMWKQITQTYWNNKPTKAQGYSGLSIKVVNSTTGPGEHLRNALWHTGNTTGQVKTLWHDPKNIGWKDF
      *********************************************************************************************************************************.***********:******

OTS   TAYRWHLIHRPRTGHIRVVMYEGKKIMADSGSIYDKTYAGGRLGLFVFSQEMVYFSDLKYECRDA
OKE   TAYRWHLIHRPRTGHIRVVMYEGKKIMADSGSIYDKTYAGGRLGLFVFSQEMVYFSDLKYECRDA
OMY   TAYRWHLIHRPRTGHIRVVMYEGKKIMADSGSIYDKTYAGGRLGLFVFSQEMVYFSDLKYECRDA
SSA   TAYRWHLIHRPRTGHIRVVMYEGKKIMADSGSIYDKTYAGGRLGLFVFSQEMVYFSDLKYECRDA
SAL   TAYRWHLIHRPRTGHIRVVMYEGKKIMADSGSIYDKTYAGGRLGLFVFSQEMVYFSDLKYECRDA
      *****************************************************************
```

OKE:MK139486
OMY:XP_021456715
OTS:XP_024234348
SAL:XP_023855494
SSA:XP_014061410

OK_TSP2

VWFC (residues 133-190)

```
OMY HMCWQDGRLFEDKEDWVVDSCTTCTCQDSKIVCHQITCPPVACASPSFIDGECCPVCL
OKE HMCWQDGRLFEDKEDWVVDSCTTCTCQDSKIVCHQITCPPVACASPSFIDGECCPVCL
OTS HMCWQDGRLFEDKEDWVVDSCTTCTCQDSKIVCHQITCPPVACASPSFIDGECCPVCL
OKI HMCWQDGRLFEDKEDWVVDSCTTCTCQDSKIVCHQITCPPVACASPSFIDGECCPVCL
SSA HMCWQDGRLFEDKEDWVVDSCTTCTCQDSKIVCHQITCPPVACASPSFIDGECCPVCL
SAL HMCWQDGRLFEDKEDWVVDSCTTCTCQDSKIVCHQITCPPVACASPSFIDGECCPVCL
    **********************************************************
```

EGF (residues 363-403)

```
OMY PIDGCLSNPCFGGVDCNSAPDGSWECGPCPLGFRGNGTHCE
OKE PIDGCLSNPCFGGVDCNSAPDGSWECGPCPLGFRGNGTHCE
OTS PIDGCLSNPCFGGVDCNSAPDGSWECGPCPLGFRGNGTHCE
OKI PIDGCLSNPCFGGVDCNSAPDGSWECGPCPLGFRGNGTHCE
SSA PIDGCLSNPCFGGVDCNSAPDGSWECGPCPLGFRGNGTHCE
SAL PIDGCLSNPCFGGVDCNSAPDGSWECGPCPLGFRGNGTHCE
    *****************************************
```

EGF (residues 462-504)

```
OMY PENPCKDKTHNCHRSSECIYISHFSEPMYKCECRIGYAGDGFI
OKE PENPCKDKTHNCHRSSECIYISHFSEPMYKCECRIGYAGDGFI
OTS PENPCKDKTHNCHRSSECIYISHFSEPMYKCECRIGYAGDGFI
OKI PENPCKDKTHNCHRSSECIYISHFSEPMYKCECRIGYAGDGFI
SSA PENPCKDKTHNCHRSSECIYISHFSEPMYKCECRIGYAGDGFI
SAL PENPCKDKTHNCHRSSECIYISHFSDPXYKCECRXGYAGDGFI
    *************************:* ****** ********
```

Type 1 (residues 196-245)

```
OMY DGWSPWSEWTECTVTCGTGTQQRGRSCDATSNTCSGPSIQTRKCSLGKCD
OKE DGWSPWSEWTECTVTCGTGTQQRGRSCDATSNTCSGPSIQTRKCSLGKCD
OTS DGWSPWSEWTECTVTCGTGTQQRGRSCDATSNTCSGPSIQTRKCSLGKCD
OKI DGWSPWSEWTECTVTCGTGTQQRGRSCDATSNTCSGPSIQTRKCSLGKCD
SSA DGWSPWSEWTECTVTCGTGTQQRGRSCDATSNTCSGPSIQTRKCSLGKCD
SAL DGWSPWSEWTECTVTCGTGTQQRGRSCDATSNTCSGPSIQTRKCSLGKCD
    **************************************************
```

Type 1 (residues 251-306)

```
OMY NGGWSLWSPWSSCSVTCGEGQITRIRHCNAPTPQLGGKDCEGQGRDTQRCEAKPCP
OKE NGGWSLWSPWSSCSVTCGEGQITRIRHCNAPTPQLGGKDCEGQGRDTQRCEAKPCP
OTS NGGWSLWSPWSSCSVTCGEGQITRIRHCNAPTPQLGGKDCEGQGRDTQRCEAKPCP
OKI NGGWSLWSPWSSCSVTCGEGQITRIRHCNAPTPQLGGKDCEGQGRDTQRCEAKPCP
SSA NGGWSLWSPWSSCSVTCGEGQITRIRHCNAPTPQLGGKDCEGQGRETQRCEAKPCP
SAL NGGWSLWSPWSSCSVTCGEGQITRIRHCNAPTPQLGGKDCEGQGRETQRCEAKPCP
    *********************************************:**********
```

Type 1 (residues 308-363)

```
OMY DGGWGPWSPWAICSATCGGGVKGRTRVCNSPEPQYGGKKCPGETNDSDACNKQECP
OKE DGGWGPWSPWAICSATCGGGVKGRTRVCNSPEPQYGGKKCPGETNDSDACNKQECP
OTS DGGWGPWSPWAICSATCGGGVKGRTRVCNSPEPQYGGKKCPGETNDSDACNKQECP
OKI DGGWGPWSPWAICSATCGGGVKGRTRVCNSPEPQYGGKKCPGETNDSDACNKQECP
SSA DGGWGPWSPWAICSATCGGGVKGRTRVCNSPEPQYGGKKCPGETNDSDACNKQECP
SAL DGGWGPWSPWAICSATCGGGFKGRTRVCNSPEPQYGGKKCPGETNDRDACNKQECP
    ********************.************************* *********
```

Type 3 (residues 507-542)

```
OMY EDSDLDGWPNQNLVCGANATYHCKKDNCPNLPNSGQ
OKE EDSDLDGWPNQNLVCGANATYHCKKDNCPNLPNSGQ
OTS EDSDLDGWPNQNLVCGANATYHCKKDNCPNLPNSGQ
OKI EDSDLDGWPNHNLVCGANATYHCKKDNCPNLPNSGQ
SSA EDSDLDGWPNQNLVCGANATYHCKKDNCPNLPNSGQ
SAL EDXDLDGWPNQELVCGANATYHCKKDNCPNLPNSGQ
    ** *******::************************
```

Type 3 (residues 543-578)

```
OMY EDFDKDGQGDACDQDDDNDGIVDERDNCPLMYNPRQ
OKE EDFDKDGQGDACDQDDDNDGIVDERDNCPLMYNPRQ
OTS EDFDKDGQGDACDQDDDNDGIVDERDNCPLMYNPRQ
OKI EDFDKDGQGDACDQDDDNDGIVDERDNCPLMYNPRQ
SSA EDFDKDGQGDACDQDDDNDGIVDERDNCPLMYNPRQ
SAL EDFDKDGQGDACRQDDDNDGIVDERDNCPLLYNPRQ
    ************ *****************:*****
```

Type 3 (residues 579-601)

```
OMY FDFDKDDVGDRCDNCPYEHNPAQ
OKE FDFDKDDVGDRCDNCPYEHNPAQ
OTS FDFDKDDVGDRCDNCPYEHNPAQ
OKI FDFDKDDVGDRCDNCPYEHNPAQ
SSA FDFDKDDVGDRCDNCPYEHNPAQ
SAL FDLMQDDVGDRCDNCPYEHNPAQ
    **: :******************
```

Type 3 (residues 602-637)

```
OMY IDTDHNGEGDACAIDIDGDEILNEQDNCPYLYNNDQ
OKE IDTDHNGEGDACAIDIDGDEILNEQDNCPYLYNNDQ
OTS IDTDHNGEGDACAIDIDGDEILNEQDNCPYLYNNDQ
OKI IDTDHNGEGDACAIDIDGDEILNEQDNCPYLYNNDQ
SSA IDTDHNGEGDVCAVDIDGDEILNEQDNCPYLYNNDQ
SAL IDTDHNGEGDACAVDIDGDEILNEQDNCPYLYNNDQ
    **********.**:**********************
```

Type 3 (residues 638-660)

```
OMY KDTDMDGVGDQCDNCPLLHNPDQ
OKE KDTDMDGVGDQCDNCPLLHNPDQ
OTS KDTDMDGVGDQCDNCPLLHNPDQ
OKI KDTDMDGVGDQCDNCPLLHNPDQ
SSA KDTDMDGVGDQCDNCPLLHNPDQ
SAL KDTDMDGVGDQCDNCPLLHNPDQ
    ***********************
```

Type 3 (residues 661-698)

```
OMY ADTDNDLVGDQCDNNQDIDEDGHQNNLDNCPYVANSNQADTDNDLVGDQ
OKE ADTDNDLVGDQCDNNQDIDEDGHQNNLDNCPYVANSNQADTDNDLVGDQ
OTS ADTDNDLVGDQCDNNQDIDEDGHQNNLDNCPYVANSNQADTDNDLVGDQ
OKI ADTDNDLVGDQCDNNQDIDEDGHQNNLDNCPYVANSNQADTDNDLVGDQ
SSA ADTDNDLVGDQCDNNQDIDEDGHQNNLDNCPYVANSNQADTDNDLVGDQ
SAL ADTDNELVGDQCDNNQDIDEDGHQNNLDNCPYVANSNQADTDNELVGDQ
    *****:*************************************:*****
```

Type 3 (residues 699-734)

```
OMY ADHDKDGKGDACDFDDDNDGIPDDRDNCRLTPNKDQ
OKE ADHDKDGKGDACDFDDDNDGIPDDRDNCRLTPNKDQ
OTS ADHDKDGKGDACDFDDDNDGIPDDRDNCRLTPNKDQ
OKI ADHDKDGKGDACDFDDDNDGIPDDRDNCRLTPNKDQ
SSA ADHDKDGKGDACDFDDDNDGIPDDRDNCRLTPNKDQ
SAL ADHDKDGKGDACDFDDDNDGIPDDRDNCRLTPNKDQ
    ************************************
```

Type 3 (residues 735-770)

```
OMY LDSDGDGRGDACKDDFDNDNIPDFLDVCPENNAVSV
OKE LDSDGDGRGDACKDDFDNDNIPDFLDVCPENNAVSV
OTS LDSDGDGRGDACKDDFDNDNIPDFLDVCPENNAVSV
OKI LDSDGDGRGDACKDDFDNDNIPDFLDVCPENNAVSV
SSA LDSDGDGRGDACKDDFDNDNIPDFLDVCPENNAVSV
SAL LDSDGDGRGDACKDDFDNDNIPDFLDVCPENNAVSV
    ************************************
```

TSP-CTER (residues 774-986)

```
OMY RKFQMVHLDPKGTAQIDPNWVVRHQGKELVQTVNSDPGIAVGFDEFNAVDFSGTFYVNTDRDDDYAGFVFGYQSSGRFYVVMWKQITQTYWEDKPSKAFGISGVSLKVVNSTTGTG
OKE RKFQMVHLDPKGTAQIDPNWVVRHQGKELVQTVNSDPGIAVGFDEFNAVDFSGTFYVNTDRDDDYAGFVFGYQSSGRFYVVMWKQITQTYWEDKPSKAFGISGVSLKVVNSTTGTG
OTS RKFQMVHLDPKGTAQIDPNWVVRHQGKELVQTVNSDPGIAVGFDEFNAVDFSGTFYVNTDRDDDYAGFVFGYQSSGRFYVVMWKQITQTYWEDKPSKAFGISGVSLKVVNSTTGTG
OKI RKFQMVHLDPKGTAQIDPNWVVRHQGKELVQTVNSDPGIAVGFDEFNAVDFSGTFYVNTDRDDDYAGFVFGYQSSGRFYVVMWKQITQTYWEDKPSKAFGISGVSLKVVNSTTGTG
SSA RKFQMVHLDPKGTAQIDPNWVVRHQGKELVQTVNSDPGIAVGFDEFNAVDFSGTFYVNTDRDDDYAGFVFGYQSSGRFYVVMWKQITQTYWEDKPSKAFGISGVSLKVVNSTTGTG
SAL RKFQMVHLDPKGTTQIDPNWVVRHQGKELVQTVNSDPGIAVGFDEFNAVDFSGTFYVNTDRDDDYAGFVFGYQSSGRFYVVMWKQITQTYWEDKPSKAFGISGVSLKVVNSTTGTG
    *************:******************************************************************************************************

OMY ENLRNALWHTGNTKNQVRTLWHDPKNIGWKDYTAYRWHLIHRPKTGFIRVVVYEGKQILADSGPVYDKTFAGGRLGLFVFSQELVFFSDLKYECRDK
OKE ENLRNALWHTGNTKNQVRTLWHDPKNIGWKDYTAYRWHLIHRPKTGFIRVVVYEGKQILADSGPVYDKTFAGGRLGLFVFSQELVFFSDLKYECRDN
OTS ENLRNALWHTGNTKNQVRTLWHDPKNIGWKDYTAYRWHLIHRPKTGFIRVVVYEGKQILADSGPVYDKTFAGGRLGLFVFSQELVFFSDLKYECRDK
OKI ENLRNALWHTGNTKNQVRTLWHDPKNIGWKDYTAYRWHLIHRPKTGFIRVVVYEGKQILADSGPVYDKTFAGGRLGLFVFSQELVFFSDLKYECRDK
SSA ENLRNALWHTGNTKNQVRTLWHDPKNIGWKDYTAYRWHLIHRPKTGFIRVVVYEGKHILADSGPVYDKTFAGGRLGLFVFSQELVFFSDLKYECRDK
SAL ENLRNALWHTGNTKNQVRTLWHDPKNIGWKDYTAYRWHLIHRPKTGFIRVVVYEGKQILADSGPVYDKTFAGGRLGLFVFSQELVFFSDLKYECRDK
    ********************************************************:***************************************:
```

OKE:MK139487
OKI:XP_020316137
OMY:XP_021432084
OTS:XP_024244025
SAL:XP_023849371
SSA:XP_014035532

OK_TSP3A

EGF (residues 277-318)

```
OTS PRSRCSPNPCFKGLSCMETSDYPGYRCSPCPEGMTGNGTHCQ
OMY PRSRCSPNPCFKGLSCMETFDYPGYRCSPCPEGMTGNGTHCQ
OKE PRSRCSPNPCFKGLSCMETFDYPGYRCSPCPEGMTGNGTHCQ
SSA PRSRCSPNPCFKGLSCMETFDYPGYRCGPCPEGMTGNGTHCQ
SAL PRSRCSPNPCFKGVSCMETFDYPGYRCGPCPEGMMGNGTHCQ
OKI PRSPCSPNPCFKGVSCMETFDYPGYRCGPCPEGMMGNGTHCQ
    *** *********:***** *******.****** *******
```

EGF (residues 319-358)

```
OTS DIDECSIAQPCYSPGACINTVKGFSCELCPPGLWGPPLFG
OMY DIDECSIAQPCYSPGACINTVKGFSCELCPPGLWGPPLFG
OKE DIDECSIAQPCYSPGACINTVKGFSCELCPPGLWGPPLFG
SSA DIDECSIAQPCYSPGACINTVKGFSCELCPPGLWGPPLFG
SAL DIDECSIAQPCYSPGACINTVKGFSCELCPPGLWGPPVFG
OKI DIDECSIAQPCYSPGACINTVKGFSCELCPPGLWGPPLFG
    *************************************:**
```

EGF (residues 373-412)

```
OTS DIDECIEVANACVPHSMCTNTIGSFRCGGCKVGYLGNQTV
OMY DIDECIEVANACVPHSMCTNTIGSFRCGGCKVGYLGNQTV
OKE DIDECIEVANACVPHSMCINTIGSFRCGGCKVGYLGNQTV
SSA DIDECIEVANACVPNSMCTNTIGSFRCGGCKVGYLGNQTV
SAL DIDECIEVANACVPHSMCTNTIGSFRCGGCKVGYLGNQTV
OKI DIDECIEVANACVPHSMCTNTIGSFRCGGCKVGYWENQTV
    **************:*** ***************  ****
```

EGF (residues 416-458)

```
OTS PRRSCATLSFNPCDANAHCIIERNGEVSCACNIGWAGNGNTCG
OMY PRRSCATLSFNPCDANAHCIIERNGEVSCACNIGWAGNGNTCG
OKE PRRSCATLSFNPCDANAHCIIERNGEVSCACNIGWAGNGNTCG
SSA PRRSCATLSFNPCDANAHCIIERNGEVSCACNIGWAGNGNTCG
SAL PRRSCATLSFNPCDANAHCIIERNGEVSCACNIGWAGNGNTCG
OKI PRRSCATLSFNPCDANAHCIIERNGEVSCACNIGWAGNGNTCG
    *******************************************
```

Type 3 (residues 459-493)

```
OTS TDTDIDGYPDRSLPCMDNDKHCKQDNCVYTPNSGQ
OMY TDTDIDGYPDRSLPCMDNDKHCKQDNCVYTPNSGQ
OKE TDTDIDGYPDRSLPCMDNDKHCKQDNCVYTPNSGQ
SSA TDTDIDGYPDRSLPCMDNDKHCKQDNCVYTPNSGQ
SAL TETDIDGYPDRSLPCMDNDKHCKQDNCVYTPNSGQ
OKI TDTDIDGYPDRSLPCMDNDKHCKQDNCVYTPNSGQ
    *:*********************************
```

Type 3 (residues 494-529)

```
OTS EDADNDGIGDQCDEDADGDGIKNVEDNCRLEPNKDQ
OMY EDADNDGIGDQCDEDADGDGIKNVEDNCRLEPNKDQ
OKE EDADNDGIGDQCDEDADGDGIKNVEDNCRLEPNKDQ
SSA EDADNDGIGDQCDEDADGDGIKNVEDNCRLEPNKDQ
SAL EEADNDGIGDQCDEDADGDGIKNVEDNCRLEPNKDQ
OKI EDADNDGIGDQCDEDADGDGIKNVEDNCRLEPNKDQ
    *:**********************************
```

Type 3 (residues 530-552)

```
OTS QNSDTDSFGDACDNCPNVPNIDQ
OMY QNSDTDSFGDACDNCPNVPNIDQ
OKE QNSDTDSFGDARDNCPNVPNIDQ
SSA QNSDTDSFGDACDNCPNVPNIDQ
SAL QNSDTDSFGDACDNCPNVPNIDQ
OKI QNSDTDSFGDACDNCPNVPNIDQ
    *********** ***********
```

Type 3 (residues 553-588)

```
OTS RDTDSNGQGDACDNDIDGDGIPNVLDNCPKVPNPMQ
OMY RDTDNNGQGDACDNDIDGDGIPNVLDNCPKVPNPMQ
OKE RDTDSNGQGDACDNDIDGDGIPNVLDNCPKVPNPMQ
SSA RDTDSNGQGDACDNDIDGDGIPNVLDNCPKVPNPMQ
SAL KDTDSNGQGDACDNDIDGDGIPNVLDNCPKVPNPMQ
OKI XDTDSNGQGDACDNDIDGDGIPNVLDNCPKVPNPMQ
     ***.*******************************
```

Type 3 (residues 589-611)

```
OTS TDRDGDGVGDACDSCPEISNPMQ
OMY TDRDGDGVGDACDSCPEISNPMQ
OKE TDRDGDGVGDACDSCPEISNPMQ
SSA TDRDGDGVGDACDSCPEISNPMQ
SAL TDRDGDGVGDACDSCPEISNPMQ
OKI TDRDGDGVGDACDSCPEISNPMQ
    ***********************
```

Type 3 (residues 612-649)

```
OTS TDIDNDLVGDVCDTDQDTDGDGHQDSRDNCPDHPNSSQ
OMY TDIDNDLVGDVCDTDQDTDGDGHQDSRDNCPDHPNSSQ
OKE TDIDNDLVGDVCDTDQDTDGDGHQDSRDNCPDHPNSSQ
SSA TDIDNDLVGDVCDTDQDTDGDGHQDSRDNCPDHPNSSQ
SAL TDIDNDLVGDVCDTDQDTDGDGHQDSRDNCPDHPNSSQ
OKI TDIDNDLVGDVCDTDQDTDGDGHQDSRDNCPDHPNSSQ
    **************************************
```

Type 3 (residues 650-685)

```
OTS LDSDNDGLGDDCDDDDDNDGFPDVQDNCRLITNPNQ
OMY LDSDNDGLGDDCDDDDDNDGFPDVQDNCRLITNPNQ
OKE LDSDNDGLGDDCDDDDDNDGFPDVQDNCRLITNPNQ
SSA LDSDNDGLGDDCDDDDDNDGFPDVQDNCRLITNPNQ
SAL LDSDNDGLGDDCDDDDDNDGIPDIQDNCRLITNPNQ
OKI LDSDNDGLGDDCDDDDDNDRIPDIQDNCRLITNPNQ
    ******************* :**:************
```

Type 3 (residues 686-721)

```
OTS KDSNSNGVGDVCENDFDNDSVWDLIDVCPESSEVTL
OMY KDSNSNGVGDVCENDFDNDSVWDLIDVCPESSEVTL
OKE KDSNSNGVGDVCENDFDNDSVWDLIDVCPESSEVTL
SSA KDSNSNGVGDVCENDFDNDSVWDLIDVCPESSEVTL
SAL KDSNSNGVGDVCENDFDNDSVWDLIDVCPESSEVTL
OKI KDSNSNGVGDVCENDFDNDSVWDLIDVCPESSEVTL
    ************************************
```

TSP-CTER (residues 725-939)

```
OTS RAYQTVILDPEGDAQIDPNWVVLNQGMEIVQTMNSDPGLAVGYTAFNGVDFEGTFHINTVTDDDYAGFIFGYQDSSSFYVVMWKQTEQTYWQSTPFRAMAQPGLQLKAVKSRTGPGEYLRNALWHTGDTNEEVKLLWSDPRNVGWR
OMY RAYQTVILDPEGDAQIDPNWVVLNQGMEIVQTMNSDPGLAVGYTAFNGVDFEGTFHINTVTDDDYAGFIFGYQDSSSFYVVMWKQTEQTYWQSTPFRAMAQPGLQLKAVKSRTGPGEYLRNALWHTGDTNEEVKLLWSDPRNVGWR
OKE RAYQTVILDPEGDAQIDPNWVVLNQGMEIVQTMNSDPGLAVGYTAFNGVDFEGTFHINTVTDDDYAGFIFGYQDSSSFYVVMWKQTEQTYWQSTPFRAMAQPGLQLKAVKSRTGPGEYLRNALWHTGDTNEEVKLLWSDPRNVGWR
SSA RAYQTVILDPEGDAQIDPNWVVLNQGMEIVQTMNSDPGLAVGYTAFNGVDFEGTFHINTVTDDDYAGFIFGYQDSSSFYVVMWKQTEQTYWQSTPFRAMAQPGLQLKAVKSRTGPGEYLRNALWHTGDTNEEVKLLWSDPRNVGWR
SAL RAYQTVILDPEGDAQIDPNWVVLNQGMEIVQTMNSDPGLAVGYTAFNGVDFEGTFHINTVTDDDYAGFIFGYQDSSSFYVVMWKQTEQTYWQSTPFRAMAQPGLQLKAVKSRTGPGEYLRNALWHTGDTNEEVKLLWSDPRNVGWR
OKI RAYQTVILDPEGDAQIDPNWVVLNQGMEIVQTMNSDPGLAVGYTAFNGVDFEGTFHINTVTDDDYAGFIFGYQDSSSFYVVMWKQTEQTYWQSTPFRAMAQPGLQLKAVKSRTGPGEYLRNALWHTGDTNEEVKLLWSDPRNVGWR
    **************************************************************************************************************************************************

OTS DKTSYRWQLSHRPQVGYIRVKLYEGTEMVADSGVVIDTTMRGGRLGVFCFSQENIIWSNLRYRCNDTVPDDFNPYRKQVLLHIKV
OMY DKTSYRWQLSHRPQVGYIRVKLYEGTEMVADSGVVIDTTMRGGRLGVFCFSQENIIWSNLRYRCNDTVPDDFNPYRKQVLLHIKV
OKE DKTSYRWQLSHRPQVGYIRVKLYEGTEMVADSGVVIDTTMRGGRLGVFCFSQENVIWSNLRYRCNDTVPDDFNPYRKQVLLHIKV
SSA DKTSYRWQLSHRPQVGYIRVKLYEGTEIVADSGVVIDTTMRGGRLGVFCFSQENIIWSNLRYRCNDTVPDDFNPYRKQVLLHIKV
SAL DKTSYRWQLSHRPQVGYIRVKLYEGTEIVADSGVVIDTTMRGGRLGVFCFSQENIIWSNLRYRCNDTVPDDFNPYRKQVLLHIKV
OKI DKTSYRWQLSHRPQVGYIRVKLYEGTEIXADSGVVIDTTMRGGRLGVFCFSQENIIWSNLRYRCNDTVPDDFNPYRKQVLLHIKV
    ***************************: *************************:******************************
```

OKE:MK139488
OKI:XP_020353535
OMY:XP_021448791
OTS:XP_024249571
SAL:XP_023844676
SSA:XP_014016151

OK_TSP3B

EGF_3 (residues 275-316)

```
OTS THSRCEPNPCYKGLACTETREYPGYRCGACPDGMTGNGTHCQ
OKE THSRCEPNPCYKGLACTETREYPGYRCGACPDGMTGNGTHCQ
OMY THSRCEPNPCYKGLACTETREYPGYRCGACPDGMTGNGTHCQ
SSA THSRCKPNPCYKGLACTETWEYPGYRCGACPDGMTGNGTHCQ
SAL PQSRCEPNLCYKGVPCTETREYPGYRCGACPEGMTGNGTHCQ
OKI PHSRCEPNLCYKGVPCTETREYPGYRCGACPEGMTGNGTHCQ
     .:***:** ****:.**** ***********:**********
```

EGF_3 (residues 317-355)

```
OTS DIDECALARPCFSPEGCVNTAKGFTCEPCPVGFSGPLLS
OKE DIDECALARPCFSPEGCVNTAKGFTCEPCPVGFSGPILS
OMY DIDECALARPCFSPEGCVNTAKGFTCEPCPVGFSGPLLS
SSA DIDECALAQPCFSPEGCVNMAKGFTCEPCPVGFSGPLLS
SAL DIDECAVARPCFSPEGCVNTAKGFTCDPCPVGFSGSLLS
OKI DIDECAVARPCFSPEGCVNTAKGFTCESCPVGFSGSLLS
    ******:*:********** ******:.*******.:**
```

EGF_3 (residues 414-456)

```
OTS LRRSCATLGFNPCDVNAHCVIKGAARVSCECNVGWAGNGHTCG
OKE LRRFCATLGFNPCDVNAHCVIKGAARVSCECNVGWAGNGHTCG
OMY LRRSCATLGFNPCDVNAHCVIKGAARVSCECNVGWAGNGHTCG
SSA LRRSCATLGFNPCDVNAHCVIKGAASVSCECNVGWAGNGHTCG
SAL LRRSCAMLGFNPCDVNAHCVIKGAAEVSCECNVGWAGNGYTCG
OKI LRRSCATLGFNPCDVNGHCVIKGAAKVSCKCNVGWAGNGYTCG
    *** ** *********.******** ***:*********:***
```

Type 3 (residues 457-491)

```
OTS PDTDIDGYPDQPLPCIDNDYHCRADNCVNTPNSGQ
OKE PDTDIDGYPDQPLPCIDNDYHCRADNCVNTPNSGQ
OMY PDTDIDGYPDQPLPCIDNDYHCRADNCVNTPNSGQ
SSA PDTDIDGYPDQPLPCIDNNYHCRADNCVNTPNSGQ
SAL PDTDSDGYPDQPLPCIDNDYHCRADNCVNTPNSGQ
OKI PDTDSDGYPDQPLPCIDNDYHCRADNCVNTPNSGQ
    **** *************:****************
```

Type 3 (residues 492-527)

```
OTS EDADGDGIGDQCDEDADGDGIKNVEDNCRLVPNKDQ
OKE EDADGDGIGDQCDEDADGDGIKNVEDNCRLVPNKDQ
OMY EDADGDGIGDQCDEDADGDGIKNVEDNCRLVPNKDQ
SSA EDADGDGIGDQCDEDADGDGIRNVEDNCCLVPNKDQ
SAL EDADGDGIGDQCDEDADGDGIQNVEDNCRLVPNKDQ
OKI EDADGDGIGDQCDEDADGDGIQNVEDNCRLVPNKDQ
    *********************:****** *******
```

Type 3 (residues 528-550)

```
OTS QNSDTDSYGDTCDNCPNVPNGSQ
OKE QNSDTDSYGDTCDNCPNVPNGSQ
OMY QNSDTDSYGDTCDNCPNVSNGSQ
SSA QNSDTDSYGDTCDNCPNVPNGSQ
SAL QNSDTDSYGDACDNCPNVPNGSQ
OKI QNSDTDSYGDACDNCPNVPNGSQ
    **********:*******.****
```

Type 3 (residues 551-586)

```
OTS KDTDGNGAGDTCDNDIDGDGIPNVLDNCPKVPNPMQ
OKE KDTDGNGAGDTCDNDIDGDGIPNVLDNCPKVPNPMQ
OMY KDTDGNGAGDTCDNDIDGDGIPNVLDNCPKVPNPMQ
SSA KDTDGNGAGDTCDNDIDGDGIPNVLDNCPKVPNPMQ
SAL KDTDGNGAGDTCDNDIDGDGIPNVLDNCPKMPNPMQ
OKI KDTDGNGAGDTCDNDIDGDGIPNVLDNCPKMPNPMQ
    ******************************:*****
```

Type 3 (residues 587-609)

```
OTS TDRDGDRVGDACDSCPEVNDPMQ
OKE TDRDGDRVGDACDSCPEVNDPMQ
OMY TDRDGDRVGDACDSCPEVNDPMQ
SSA TDRDGDRVGDACDSCPEVNDPMQ
SAL TDRDGDGVGDACDSCPEVNDPLQ
OKI TDRDGDGVGDACDSCPEVNDPLQ
    ****** **************:*
```

Type 3 (residues 610-647)

```
OTS SDTDNDLVGDVCDTNQDMDGDGHQDSRDNCPDVPNSSQ
OKE SDTDNDLVGDVCDTNQDMDGDGHQDSRDNCPDVPNSSQ
OMY SDTDNDLVGDVCDTNQDMDGDGHQDSRDNCPDVPNSSQ
SSA SDIDNDLVGDVCDNNQDMDGDGHQDSRDNCPDVPNSSQ
SAL SDMDNDLVGDVCDTNQDMDGDGHQDSRDNCPDVPNSSQ
OKI SDMDNDLVGDVCDTNQDMDGDGHQDSRDNCPDVPNSSQ
    ** **********.************************
```

Type 3 (residues 648-687)

```
OTS LDSDNDGIGNECDDDDDNDGIPDARPPGPDNCRLIPNPSQ
OKE LDSDNDGIGNECDDDDDNDGIPDARPPGPDNCRLIPNPSQ
OMY LDSDNDGIGNECDDDDDNDGIPDARPPGPDNCRLIPNPSQ
SSA LDSDNDGIGNECDDDDDNDGIPDARPPGPDNCRLIPNPSQ
SAL LDSDNDGIGDECDDDDDNDGIPDAGPPGPDNCRLIPNPSQ
OKI LDSDNDGIGDECDDDDDNDGIPDTGPPGPDNCRLIPNPSQ
    *********:*************: ***************
```

Type 3 (residues 688-723)

```
OTS TDTNGNGIGDMCENDFDNDSVIDLIDVCPESAEVTM
OKE TDTNGNGIGDMCENDFDNDSVIDLIDVCPESAEVTM
OMY TDTNGNGVGDMCENDFDNDSVIDLIDVCPESAEVTM
SSA TDTDGNGVGDMCENDFDNDSVIDLIDVCPESAEVTM
SAL TDTDRNGIGDMCENDFDNDSVIDLIDVCPESAEVTM
OKI TDTDRNGIGDMCENDFDNDSVIDLIDVCPESAEVTM
    ***: **:****************************
```

TSP-CTER (residues 727-941)

```
OTS RNYQTVILDPEGDAQIEPKWIVLNRGMEIVQTMNSDPGLAIGYTAFNGVDFEGTFHINTATDDDYVGFIFGYQDSSSFYVVMWKQTEQTYWQSLPFRAMAQPGLQLKAVKSRTGPGEYLRNALWHTGDTPGEVTLLWKDPRNVGWRDKTSYR
OKE RNYQTVILDPEGDAQIEPKWIVLNRGMEIVQTMNSDPGLAIGYTAFNGVDFEGTFHINTATDDDYVGFIFGYQDSSSFYVVMWKQTEQTYWQSLPFRARAQPGLQLKAVKSRTGPGEYLRNALWHTGDTPGEVTLLWKDPRNVGWRDKTSYR
OMY RNYQTVILDPEGDAQIEPKWIVLNRGMEIVQTMNSDPGLAIGYTAFNGVDFEGTFHINTATDDDYVGFIFGYQDSSSFYVVMWKQTEQTYWQSLPFRAMAQPGLQLKAVKSRTGPGEYLRNALWHTGDTPGEVTLLWKDPRNVGWRDKTSYR
SSA RNYQTVILNPEDDAQIEPKWIVLNRGMEIVQTMNSDPGLAIGYTAFNGVDFEGTFHINTATDDDYVGFIFGYQDSSSFYVVMWKQTEQTYWQSLPFRAMAQPGLQLKAVKSHTGPGEYLRNALWHTGDTPGEVTLLWKDPRNVGWRDKTSYR
SAL RAYQTVILDQEGEAQIDPNWIVLNRGMEIVQTMNSDPGLAIGITAFNGVDFEGTFHINTATDDDYVGFIFGYQDSSSFYVVMWKQMEQTYWQSLPFRAIAQPGLQLKAVKSRTGPGEYLRNALWHTGDTPGEVTLLWKDPRNVGWRDKTSYR
OKI RAYQTVILDQEGEAQIDPNWIVLNRGMEIVQTMNSDPGLAIGVTAFNGVDFEGTFHINTATDDDYVGFIFSYQDSSSFYVVMWKQMEQTYWQSLPFRAMAQPGLQLKAVKSRTGPGEYLRNALWHTGDTPGEVTLLWKDARNVGWRDKTSYR
    * ******: *.:***:*:*********************** **************************.************** ************ ************:***************************.************

OTS WHLSHRPQMGYIKVRLYEGMTLVADSGVLVDTSMRGGRLGVFCFSQEQIIWSNLGYRCNDTVPEDYEFYRKQIHIRV
OKE WHLSHRPQMGYIKVRLYEGMTLVADSGVLVDTSMRGGRLGVFCFSQEQIIWSNLGYRCNDTVPEDYEFYRKQIHIRV
OMY WHLSHRPQMGYIKVRLYEGMTLVADSGVLVDTSMRGGRLGVFCFSQEQIIWSNLGYRCNDTVPEDYEFYRKQIHIRV
SSA WHLSHRPQVGYIKVRLYEGMTLVADSGVLVDTSMRGGRLGVFCFSQEQIIWSNLGYRCNDTVPEDYEFYHKLIHIRV
SAL WHLSHRPQVGYIRVRLYEGMTLVADSGVVVDTSMRGGRLGVFCFSQEQIIWSNLGYRCNDTVPEDYEFYRKQIHVRV
OKI WHLSHRPQVGYIRVRLYEGMTLVADSGVVVDTSMRGGRLGVFCFSQEQIIWSNLGYRCNDTVPEDYEFYRKQMHIRV
    ********:***:***************:****************************************:* :*:**
```

OKE:MK139489
OKI:XP_020362668
OMY:XP_021416042
OTS:XP_024251431
SAL:XP_023832323
SSA:XP_013997754

OK_TSP4B

EGF_3 (residues 275-315)

```
OTS GPSKCSPGMCFNQDMCIPAEGGRFTCAPCPDGYTGDGVHCD
OKI GPSKCSPGMCFNQDMCIPAEGGRFTCAPCPDGYTGDGVHCD
OKE GPSKCSPGMCFNQDMCIPAEGGRFTCAPCPDGYTGDGVHCD
OMY GPSKCSPGMCFNQDMCIPAEGGRFTCAPCPDGYTGDGVHCD
SAL GPFQCSPGMCFNQDMCIPAEGGRFTCAPCPDGYTGDGVHCD
SSA GPSQCSPGMCFNQDMCIPAEGGGFTCAPCPDGYTGDGVHCD
    ** :****************** ******************
```

EGF_3 (residues 316-353)

```
OTS DVNECQFNPCFPGVRCVNTAPGFRCERCPLGYTGLEIN
OKI DVNECQFNPCFPGVRCVNTAPGFRCERCPLGYTGLEIN
OKE DVNECQFNPCFPGVRCVNTAPGFRCERCPLGYTGLEIN
OMY DVNECQFNPCFPGVRCVNTAPGFRCERCPLGYTGLEIN
SAL DVNECQFKPCFSGVRCVNTAPGFRCERCPLGYTGLEIN
SSA DVNECQFNPCFPGVRCVNTAPGFRCERCPLGYTGLEIN
    *******:***.**************************
```

EGF_3 (residues 369-410)

```
OTS DIDECQGPPDNGGCTANSHCHNTRGSFRCGECKSGFSGDQVS
OKI DIDECQGPPDNGGCTANSHCHNTRGSFRCGECKSGFSGDQVS
OKE DIDECQGPPDNGGCTANSHCHNTRGSFRCGECKSGFSGDQVS
OMY DIDECQGPPDNGGCTANSHCHNTRGSFRCGECKSGFSGDQVS
SAL DIDECQGPPDNGGCTANSHCHNTRGSFRCGECKSGFSGDQVS
SSA DIDECQGPPDNGGCTANSHCHNTRGSFRCGECKSGFSGDQVS
    ******************************************
```

EGF_3 (residues 414-456)

```
OTS GGRLCGNGQPNPCDSNAQCVVERDGSVSCECDIGWAGNGYVCG
OKI GGRLCGNGQPNPCDSNAQCVVERDGSVSCECDIGWAGNGYVCG
OKE GGRLCGNGQPNPCDSNAQCVVERDGSVSCECDIGWAGNGYVCG
OMY GGRLCGNGQPNPCDSNAQCVVERDGSVSCECDIGWAGNGYVCG
SAL GERLCGNGQPNPCDSNAQCVVERDGSVSCHCDIGWAGNGYVCG
SSA GERLCGNGQPNPCDSNAQCVVERDGSVSCQCDIGWAGNGYVCG
    * ***************************.*************
```

Type 3 (residues 457-489)

```
OTS KDTDIDAYPDDKLRCRDNNCKKDNCMFVPNSGQ
OKI KDTDIDAYPDDKLRCRDNNCKKDNCMFVPNSGQ
OKE KDTDIDAYPDDKLRCRDNNCKKDNCMFVPNSGQ
OMY KDTDIDAYPDDKLRCRDNNCKKDNCMFVPNSGQ
SAL KDTDIDAYPDEKLRCRDNNCKKDNCAFVPNSGQ
SSA KDTDIDAYPDEKLRCRDNNCKKDNCMFVPNSGQ
    **********:************** *******
```

Type 3 (residues 490-525)

```
OTS EDADRDGLGDACDDDADSDGIVNIEDNCWLHPNVNQ
OKI EDADRDGLGDACDDDADSDGIVNIEDNCWLHPNVNQ
OKE EDADRDGLGDACDDDADSDGIVNIEDNCWLHPNVNQ
OMY EDADRDGLGDACDDDADSDGIVNIEDNCWLHPNVNQ
SAL EDADRDGLGDACDDDADSDGIVNIEDNCWLHPNVNQ
SSA EDADRDGLGDACDDDADSDGIVNIEDNCWLHPNVNQ
    ************************************
```

Type 3 (residues 526-548)

```
OTS KNSDKDLHGDVCDNCLTTENPDQ
OKI KNSDKDLHGDACDNCLTTENPDQ
OKE KNSDKDLHGDACDNCLTTENPDQ
OMY KNSDKDLHGDACDNCLTTENPDQ
SAL KNSDKDLHGDACDNCLTTENPDQ
SSA KNSDTDLHGDACDNCLTTENPDQ
    ****.*****.************
```

Type 3 (residues 549-584)

```
OTS RDTDKDGLGDNCDDDMDGDLKNILDNCQRVANLDQ
OKI RDTDKDGLGDDCDDDMDGDLKNILDNCQRVANLDQ
OKE RDTDKDGLGDDCDDDMDGDLKNILDNCQRVANLDQ
OMY RDTDKDGLGDDCDDDMDGDLKNILDNCQRVANLDQ
SAL RDTDKDGLGDDCDDDMDGDLKNILDNCQRVTNLDQ
SSA RDTDKDGLGDDCDDDMDGDLKNILDNCQRVANLDQ
    **********:*******************:****
```

Type 3 (residues 585-607)

```
OTS RDRDNDGVGDACDXCPDMVNPNQ
OKI RDRDNDGVGDACDSCPDMVNPNQ
OKE RDRDNDGVGDACDSCPDMVNPNQ
OMY RDRDNDGVGDACDSCPDMVNPNQ
SAL RDRDNDGVGDACDSCPDMVNPNQ
SSA RDRDNDGVGDACDSCPDMVNPNQ
    ************* *********
```

Type 3 (residues 608-645)

```
OTS SDVDDDLVGDTCDTNIDSDGDGHQNTKDNCPTVINSSQ
OKI SDVDDDLVGDTCDTNIDSDGDGHQNTKDNCPTVINSSQ
OKE SDVDDDLVGDTCDTNIDSDGDGHQNTKDNCPTVINSSQ
OMY SDVDDDLVGDTCDTNIDSDGDGHQNTKDNCPTVINSSQ
SAL SDVDDDLVGDTCDTNIDSDGDGHQNTKDNCPTVINSSQ
SSA SDVDDDLVGDTCDTNIDSDGDGHQNTKDNCPTVINSSQ
    **************************************
```

Type 3 (residues 646-681)

```
OTS LDTDKDGQGDECDDDDDNDGILDQADNCRLVVNPDQ
OKI LDTDKDGQGDECDDDDDNDGILDQADNCRLVVNPDQ
OKE LDTDKDGQGDECDDDDDNDSILDQADNCRLVVNPDQ
OMY LDTDKDGQGDECDDDDDNDGILDQADNCRLVVNPDQ
SAL LDTDKDGQGDECDDDDDNDGILDQADNCRLVVNPDQ
SSA LDTDKDGQGDECDDDDDNDGILDQADNCRLVVNPDQ
    *******************.****************
```

Type 3 (residues 682-717)

```
OTS TDEDNDGVGDACAGDFDQDKVIDRIDNCPENAEVTL
OKI TDEDNDGVGDACAGDFDQDKVIDRIDNCPENAEVTL
OKE TDEDNDGVGDACAGDFDQDKVIDRIDNCPENAEVTL
OMY TDEDNDGVGDACAGDFDQDKVIDRIDNCPENAEVTL
SAL TDEDNDGVGDACAGDFDQDKVIDRIDNCPENAEVTL
SSA TDEDNDGVGDACAGDFDQDKVIDRIDNCPENAEVTL
    ************************************
```

TSP-CTER (residues 721-935)

```
OTS RAYQTVVLDPEGDAQIDPNWVVLNQGMEIVQTMNSDPGLAVGYTAFSGVDFEGTFHVNTVTDDDYAGFIFGYQDSSSFYVVMWKQTEQTYWQAVPFRAVAEPGIQLKAVKSKTGPGEYLRNSLWHTGDTNDQVRLLWKDKR
OKI RAYQTVVLDPEGDAQIDPNWVVLNQGMEIVQTMNSDPGLAVGYTAFSGVDFEGTFHVNTVTDDDYAGFIFGYQDSSSFYVVMWKQTEQTYWQAVPFRAVAEPGIQLKAVKSKTGPGEYLRNSLWHTGDTNDQVRLLWKDKR
OKE RAYQTVVLDPEGDAQIDPNWVVLNQGMEIVQTMNSDPGLAVGYTAFSGVDFEGTFHVNTVTDDDYAGFIFGYQDSSSFYVVMWKQTEQTYWQAVPFRAVAEPGIQLKAVKSKTGPGEYLRNSLWHTGDTKDQVRLLWKDKR
OMY RAYQTVVLDPEGDAQIDPNWVVLNQGMEIVQTMNSDPGLAVGYTAFSGVDFEGTFHVNTMTDDDYAGFIFGYQDSSSFYVVMWKQTEQTYWQAVPFRAVAEPGIQLKAVKSKTGPGEYLRNSLWHTGDTNDQVRLLWKDKR
SAL RAYQTVVLDPEGDAQIDPNWVVLNQGMEIVQTMNSDPGLAVGYTAFSGVDFEGTFHVNTMTDDDYAGFIFGYQDSSSFYVVMWKQTEQTYWQAVPFRAVAEPGIQLKAVKSKTGPGEYLRNSLWHTGDTNDQVRLLWKDKR
SSA RAYQTVVLDPEGDAQIDPNWVVLNQGMEIVQTMNSDPGLAVGYTAFSGVDFEGTFHVNTVTDDDYAGFIFGYQDSSSFYVVMWKQTEQTYWQAVPFRAVAEPGIQLKAVKSKTGPGEYLRNSLWHTGDTNDQVRLLWKDKR
    ***********************************************************:*********************************************************************:***********

OTS NVGWKDKVSYRWYLQHRPQVGYIRARFYEGPNLVADSGVKIDNSMRGGRLGVFCFSQENIIWSNLKYRCNDTIPEDYQEYSAQNTE
OKI NVGWKDKVSYRWYLQHRPQVGYIRARFYEGPNLVADSGVKIDNSMRGGRLGVFCFSQENIIWSNLKYRCNDTIPEDYQEYSAQNTE
OKE NVGWKDKVSYRWYLQHRPQVGYIRARFYEGPNLVADSGVKIDNSMRGGRLGVFCFSQENIIWSNLKYRCNDTIPEDYQEYSAQNTE
OMY NVGWKDKVSYRWYLQHRPQVGYIRARFYEGPNLVADSGVKIDNSMRGGRLGVFCFSQENIIWSNLKYRCNDTIPEDYQEYSAQNTE
SAL NVGWKDKVSYRWYLQHRPQVGYIRARFYEGPNLVADSGVKIDNSMRGGRLGVFCFSQENXIWSNLKYRCNDTIPEDYQEYSAQNTE
SSA NVGWKDKVSYRWYLQHRPQVGYIRARFYEGPNLVADSGVKIDNSMRGGRLGVFCFSQENIIWSNLKYRCNDTIPEDYQEYSAQNTE
    *********************************************************** **************************
```

OKE:MK139490
OKI:XP_020328796
OMY:XP_021461878
OTS:XP_024273488
SAL:XP_023857689
SSA:XP_014026236
