# Supplementary material for: Dataset for characterization of thrombospondin family in chum salmon (Oncorhynchus keta)
Source: Data Brief. 2019 Jan 9;22:866–70. doi: 10.1016/j.dib.2019.01.008 (PMC6362859; doi:10.1016/j.dib.2019.01.008)
Supplement: Supplementary file 3 — Supplementary material Supplementary B. Multiple alignment of chum salmon TSP cDNA isoforms. [file mmc3.pdf]

>Multiple alignment of chum salmon TSP cDNA isoforms

```
TSP3A     ------------------------------------------------------------------------------------------------------------------------------------------------------------------------------------
TSP3B     ------------------------------------------------------------------------------------------------------------------------------------------------------------------------------------
TSP4B     ------------------------------------------------------------------------------------------------------------------------------------------------------------------------------------
TSP1      ATGAAGTTGACAGGAATATTTCTGCTGTTGATGCTTTTGACCTGCGAGAGCGTACGAGTTGCAGAGAACCGAGACGACAATAGCGTGTACGACCTGTTCGAACTGGTCCAAGTCTCCAAGAAGAACCACGGAGTGACCCTGGTGAAAGGCGACGACCCATACAGTCCCGCCTACAAGATC
TSP2      ------------------------------------------------------------------------------------------------------------------------------------------------------------------------------------

TSP3A     ------------------------------------------------------------------------------------------------------------------------------------------------------------------------------------
TSP3B     ------------------------------------------------------------------------------------------------------------------------------------------------------------------------------------
TSP4B     ------------------------------------------------------------------------------------------------------------------------------------------------------------------------------------
TSP1      CTCAACCCGGACCTGATCCCCGAGCTCCCCGAGAGCTCCTTCAGGGACCTCATCGATTCCATCCATGCCGAGAGGGGCTTCCTCCTCCTGCTTAACTTCAAGCAGTTTAAGCGGACCAGGGGCTCCCTCTTGACCGTGGAGGAGCGGGACGGATCAGGAGCCGTGTTCGAGATTGTCTCG
TSP2      ------------------------------------------------------------------------------------------------------------------------------------------------------------------------------------

TSP3A     ------------------------------------------------------------------------------------------------------------------------------------------------------------------------------------
TSP3B     ------------------------------------------------------------------------------------------------------------------------------------------------------------------------------------
TSP4B     ------------------------------------------------------------------------------------------------------------------------------------------------------------------------------------
TSP1      AATGGAAAAGCGAACACCCTGGATGTGGTTTTCTCCACCGTTAACAAGCAACAGGTGGTGTCGATAGAAGATGTGGACTTGGCGACGGGCCACTGGAAGAATATTACGCTGTTCGTGCAGGAGGACCGGGCGCAGCTGTATGTTGGCTGTGAGGAGGTGAACACGGCGGAACTGGACGCG
TSP2      ------------------------------------------------------------------------------------------------------------------------------------------------------------------------------------

TSP3A     -------------------------------------------------------------------------------------------------------------------------ATGGGTGCGAGGCGCGATGTGCCAACTTTTCTGGCTCTATCCACATTTCTATTCATTGC
TSP3B     -------------------------------------------------------------------------------------------------------------------------ATGGATATGGTGAGGTTTGTGCTAAATTTGCTGCTTGCGAGCGCGGTGGTTCTCTTGG-
TSP4B     -------------------------------------------------------------------------------------------------------------------------------ATGGGGGTGTGGATCAGAAAAGTGGTCCTCTCTCTGCTGATGCTACAACTGGC
TSP1      CCCATTCAGAGCATCCTCACTCAGGAGACTCCCGCCACAGCGCGCCTCAGGATCGGGAAGGGAGCGGTGAAAGACCGGTTCATGGGGGTGCTCCAAAACGTGCGCTTTGTCTTTGGAACCACGTTGGACGCGATTCTGCGCAACAAGGGATGTCAAAACTCTGTT-CTGACGGA--TGAG
TSP2      -----------------------------------------ATGTTCGTCGCCAAGGGATCCATCCGCGAAA-ACCATTTCAGGGGCTTACTGCAGAATGTTCGCTTCATGTTCGACACATCCGTGGATGATGTTCTGAGGAACAAAGGCTGTGAAGTCACTAAGGCTGACGAGGCCAAT
                                                                                                                                            *              **     *               *

TSP3A     AACATG-TGAGCCAATGGATAAGAAAGATATGCAAGTCATTGACATGCTAACGCTG---GACTCCAAGACCAGTGTGTCTGCGGTGGAGAAGGTGACAGGTGCCATGAGTGTGCTCAGTGACATCTACATCGTGTCCACATTCCGCCTGCCTCCCAAGATGGGAGGGGTGCTGCTGGGCC
TSP3B     ---GCT-TGA-CCAGGGCTTCAGAAA----TGCAAGTGATTGACATGCTGGAGCTGCACGATGCCAGGCAGAGTGCGTCCGCGGTGGGGAAGATGTCGGGGGCTCTGGGCTTCGTGTCGGACCTTTACCTGGTGTCAACTCTTCGTCTGCCGCCCAAACTGGGAGGAGTGTTGTTGGGAG
TSP4B     AACGAC-TG--TCACAGCACAAGGCA-------TTGTGTACGACCTGCTGGTGTCTCCAGACTGCCTGCCTGACCTGACGCAGGGAGGGCTGAAGAATAAAGGTCTGGAT---------GAGGCCTTCCTCCTCTCCTCCTTCAGGCTCCATAGCAAGTCCCCCTCTCATCTCTACAGCA
TSP1      ATCGTCCTTATCGACGACACCATCA----------ATGGGTCCAGCC-CTGCCATTAGGACGGACTACACTGGCCACAAAACTAAAGACCTG----CAGATGATCTGTGGGTTCT--CCTGTGAGGACCTGGCTGGCATGTTCAAGGAACTGAAGGGGTTGGGAGTGGTGGTGAAGGAGC
TSP2      GTGGTGGTGAGTGAGAGCACAGATATGG--AGGATGTGAGTACCTCCACAGCCATCACCACTAACTTCATCGGCCAAAAGACAGAGAAGCTGGGAGCAGAT-ATGTGTGAACGAT--CCTGTGAGGAACTCAGCACCATGTTCCAGGAGCTCAAAGGACTGCGGGTTGTAGTGGGGAACC
                 *     *                      *                           *                          *             **                      *           *       *                     *

TSP3A     TCTACAGCAAGGAGGAGAACAAGAAGTACCTGGAGCTGGCCATCATGGGAAAGATCAATAAAGCTCTGGTGCGTTACGTGAGGGAGGATGGCAAAATTCACACAGTGAACCTCCAGAGCACAAAC--CTGGCTGATGGTCGCACACACTCCATTATTCTCCGGATCGGAGGCCTGCGCAG
TSP3B     TCTACAACAAAGGCGACAACAGGAAGTACCTGGAGGTGGCCCTCATGGGCAAGGTCAACAAAGTTCTGGTGCGCTACGTACGCGAGGATGGTAAAATACACACTGTGAACCTCCAGAACCCTGGC--CTCTCCGATGGACGCACCCAGTCACTCATCCTCCGTGTGGGTGGGCTCCAACG
TSP4B     TCATCAACCCCAGGGACAACAGCAAGTACCTGGAGTTCACCCTGCAGGCCAAACTCAACAAGGTGACGATCCGTTACCAGAAGACTGATGGTAGGGCTGGCACCACCAGTTTTAGCCACCCCTCT--CTGGCGGACGGCAAGGAGCATCATGTGATGATCCATGCCAGTGGTCTGCAGAG
TSP1      TGTCTAACGAGCTGCGCAAAGTGACGGACGAGAACAAGTTTTTGATGAACAGAGTTGGGA-----------TCCACAGTGGAG----TCTGTCTGCACAACGGCATCGTCCACAAGAACAAGGCCGAGTGGACCGTCGACGACTGTACCGAGTGCACTTGTCAAAACTCTGCCACCGTGT
TSP2      TCATAGACGGCCTGCAGAAAGTGACAGAGGAGAACACAGTAATGAAGGATGCTTTGGGGAAGATGAAGAACTCCAAGGAGAAACACATGTGTTGGCAGGACGGACGGCTGTTTGAAGATAAGGAGGACTGGGTGGTGGACAGCTGCACCACGTGTACCTGTCAGGACTCAAAGATCGTGT
          *      *         **    *   *   * *        *   *       *    *                              *         *                           *        *        *     *     *                *

TSP3A     AGACAACCTACACCTGGAGCTCTACGTCAATTGTCGATTGGCTGACTCCAGCCAGGGCCTCCCTCCATTGGTCCCT--CTCCCCGCAGAGAAGG------TGGAGATTCGTAATGGCTTCAAGGCCTACGCAAGGCTACAGGGTGCTGTGGAGTCCCTCAAAATTGCACTAGGGGGCAGT
TSP3B     AAACCACCTCCACATGGAACTCTATGTCAACTGCCGATTGGCTGACTCAGCACAGGTCCTGCCATCCATGGTCCAG--CTCCCAGCCGAGGCAGAGTCTGTGGAGATACGCAACGGGCAGAAGGCCTATGCCAGGCTACAGGGCTCAGTGGACTCTTTGAAACTGGCTTTGGGTGGCTCT
TSP4B     AGGACCAGGTCGTATGGCGGTCTACGTAGACTGTAGACTGGCTCACATTGTGGATGAGCTGCCTGCTGCCTTCGGG--TCGCTGACACCTGGACACAACAAGGTGGCGCTTAGGACCCTGCAGCCC---ACCGGGCAGGATGAGTTGACGGA---CCTGAAACTGGTGATAGAGGACACG
TSP1      GCCGTAAGATCTCCTGCCCCCTGATCCCGTGTGCCAACGCAACTGTGCCCGATGGAGAGTGCTGCCCGCGCTGTGGAACACCGAGTGACTACGCGGAGGATGGATGGTCCCTGTGGTCTGAATGGACCCACTGTTCCGTGTCTTGTGGGCGGGGAATTCAGCAGAGAGGCCGCTCCTGCG
TSP2      GTCACCAAATCACTTGTCCTCCTGTGGCCTGTGCTAGCCCCTCTTTCATCGACGGAGAATGTTGCCCTGTGTGT------CTGTCTAAGGACAGTGAGGACGGCTGGTCCCCCTGGTCGGAGTGGACAGAGTGTACTGTCACCTGTGGGACAGGTACTCAACAGAGGGGACGCTCATGTG
                    *   **               **                          *     *     *                             **     *                          *                     * *     *     *

TSP3A     GTGGCCAAAGCAGGTACCCTGACAGACTGTCCATTCCAGG-GGGATTCATCAGTC--TACAGCACAGTCGGTACGACT-GCAG--AAGTGAAC----TCCAT-----TCTAGGAG----ATCACACAAAGGCTCTGATTG-GTCAGCTGATCA--TCTTTAACCAGATCCTAGGAGA---
TSP3B     ATAGCCAAAGCAGGAATCCTTACCGACTGTCCGTTTCAGG-GAGATACACATTTT--CAGAATTCAGTTAGC-------GCCG--ATGTCAAT----GCCAT-----TCTGGGTG----ACCACACCAAGGCTTTGATTG-GCCAGCTGATCA--TCTTCAACCAGATTCTTGGTGA---
TSP4B     TTAGATAACGTCGCTACGCTCCAGGACTGCGGCAGACAGC-AGAGCGAGTCTCTGGGTACTATTCAGCTACTGGGCCT-CCAGGCAGGTGGACAGGATCCAT-----CTCAGATGTTAGAGCTCCACAAGATGATGTCAGAGATGAAGGACCTGCTCCTCCAGCAGATTA-AGGAGACCA
TSP1      ACCGCATCAACAACAACTGCGAGGGAACCTCGGTCCAAACCAGGGACTGCTACCTTCAGGAGTGTGACAAACGTTTCAAGCAGGACGGTGCCTGGAGCCACTGGTCCCCCTGGTCATCCTGCTCGGTCACCTGTGGGGCGGGTGTCATCACCCGTATCCGCCTCTGCAACTCCCCCACAC
TSP2      ATGCCACCAGTAACACCTGCTCCGGACCATCCATCCAGACCAGGAAATGCAGCCTGGGAAAATGTGACAGCCGAGTTCGTCAGAACGGTGGGTGGAGCCTGTGGTCTCCCTGGTCATCATGCTCAGTGACGTGTGGCGAGGGCCAGATCACCAGGATACGCCACTGCAACGCACCCACAC
                                  **                                                      * *    **         *  *         *         * *    *      *   * *       * *           * *          *
```

```
TSP3A    -GCTGCGAGAGGACATCAGAGAGCAGGTGAAGGAGATGTC-CCTGATTAGGAACACCATTCTGGAGTGCCAAGTGTGTGGCTTCCAAGACCCTCGTTCCCGCTG------CTCCCCCAACCCCTGTT---------TTAAGGGCCTGTCCTGCATGGAGACCTTTGACTATCCAGGGTAC
TSP3B    -ACTCCGAGAGGACATCAGGGAACAGGTAAAGGAGATGTC-TCTGATTAGAAACACTATCCTGGAGTGCCAAGTGTGTGGCTTCCATGAGACCCACTCTCGCTG------CGAGCCCAACCCCTGCT---------ATAAGGGCTTGGCCTGTACAGAGACCAGGGAGTACCCAGGGTAC
TSP4B    CATTCCTCAGAAACACCATCTCA-GAGTGTCTGGCATGTGGTCTGGGTAGACAACATGGCGTCAGG--CCCAGGTACAGGTCCACGGTCAGGCCCG-TCCAAGTG------TAGTCCAGGTATGTGTT---------TCAATCAGGACATGTGTATCCCGGCCGAGGGAGGAC---GCTTC
TSP1     CCCAGCTGGGAGGCAAGGACTGCCAAGGAGAGGGAAGACAGACTGAGACGTG-CACCAAGTCACCCTGCCCAATCAACGGTAACTGGGGACCCTGGTCGCTATGGGATACCTGCTCTGCCACCTGTGGGGGAGGAGCTCAGACACGTAAACGTCTCTGCAACGACCCGCCCCTAAATAT
TSP2     CACAACTGGGGGGCAAGGACTGTGAGGGCCAGGGGAGAGATACACAGCGCTG-TGAGGCCAAGCCCTGTCCCATTGACGGTGGTTGGGGACCGTGGTCGCCATGGGCGATCTGCTCGGCAACCTGTGGGGGTGGAGTTAAGGGTCGGACCCGTGTCTGTAACAGCCCCGAACCCCAGTAC
               *       **          *     *  *     *                      *   *    *           *    **    **         *       **            *          *         *         *     *

TSP3A    CGCTGTAGTCCCTGTCCGGAGGGCATGACGGGCAACGGAACCCACTGCCAAGACA------TTGATGAGTGTTCCATAGCCCAGCCCTGCTACTCTCCAGGTGCGTGTATAAACACAGTGAAGGGTTTCAGC---TGTGAGCTCTGCCCCCCTGGTCTCTGGGGCCCACCCCTCTTTGGG
TSP3B    CGTTGTGGTGCCTGCCCAGACGGCATGACGGGGAATGGCACCCACTGCCAGGACA------TTGACGAGTGTGCTCTAGCCCGGCCCTGTTTCTCGCCCGAGGGTTGCGTAAACACGGCAAAGGGCTTCACC---TGTGAACCATGTCCCGTGGGCTTCTCCGGACCCATCCTTAGCGGG
TSP4B    ACCTGCGCCCCCTGCCCCGACGGATACACTGGAGACGGAGTTCACTGTGATGACG------TGAACGAGTG---CCAGTTTAACCCCTGTTTCCCCGGCGTGAGGTGTGTGAACACAGCTCCTGGGTTCCGC---TGTGAGAGATGCCCTTTGGGATACACTGGTCTTGAGATCAACGGA
TSP1     GGTGGTAAGGAGTGCCAGGGTGACTCCAAGGCCACTCAGCAGTGCAACAAGAACGCCTGTCCCATCGATGGCTGTCTGTCCAACCCTTGCTTCCCTGGGACCAAGTGCACCAGCTTCCCTGATGGAACATGGAGGTGTGGAAAATGTCCCTCTGCTTACTCTGGTGACGGCATCAAGTGC
TSP2     GGAGGCAAGAAGTGCCCTGGGGAGACCAACGATAGTGATGCCTGCAACAAACAAGAATGCCCTATCGACGGTTGTCTGTCTAACCCATGCTTCGGAGGAGTGGACTGTAACAGTGCCCCCGACGGATCCTGGGAGTGTGGGCCATGCCCTCTCGGTTTCCGCGGCAACGGAACCCACTGC
              *       ** *  *  *      *  *            *    *  *            **  *            ** ** * *           **    *           **          ****     ** **     *    *   **              *

TSP3A    GTCGGACT-GGAGTACGCCAACCACCA-CAAACAGGAGTGTGCAGAC--ATCGATGAGTG------TATTGAAGTTGCCAACGCCTGTGTGCCCCACTCCATGTGTATCAACACCATCGG-CTCGTTCAGGTGTGGTGGCTGTAAGGTGGGTTACTTGGGGAACCAGACAGTGGGCTGCG
TSP3B    CTTGGGGT-GGAGTTTGCCAAGAGCCA-CAAGCAGAAGTGCAGAGAT--GTGGATGAGTG------TGCTGTCCTCTACAAGGCCTGTGTGCCGAACTCTGTCTGCATCAACACAGAGGG-CTCGTTCAGGTGTGGTCCATGTAAGGAGAGCTTCGTAGGCAACCAGACGGTGGGCTGTT
TSP4B    GTGGGAGT-GTCCTACGCACAGTCACA-CAAACAGGTGTGTGATGAC--ATAGATGAATGCCAGGGTCCTCCAGACAACGGAGGCTGCACCGCCAACTCTCACTGCCACAACACCAGGGG-TTCATTCCGTTGTGGGGAGTGTAAGAGTGGCTTCTCTGGGGACCAGGTGAGCGGCTGCA
TSP1     AAGGACATTGACGAGTGCAAAGAGGTTCCTGACGCTTGCTTCGTGTTCAACGGAGTCCACCGCTGTGAGAACACTGACCCTGGTTACAACTGTCTGCCCTGTCCCGCACGCTACTCTGGACCTCAGCCCTTCGGCAGGGGAGTGGAGGAGGCCAC---CGCCAAGAAACAG--GTGTGTA
TSP2     GAAGACGTCAACGAGTGTGACATGGTGTCTGACATATGCTTTAAAGCTGGTGGAGCATCGCGTTGCATCAACACAGACCCAGGGTTCCACTGTCTGTCCTGCCCACCTCGCTACAAGGGAACGCAGCCCTTCGGCATGGGGGTGGAAGCTGCCAA---GAAGAACAAACAG--GTCTGCG
               *   *        *           *    *    *              **                        *   *                 *         *   **     *     *    *     *         **         *             *  *        *  **

TSP3A    TGCCCCGTAGGTCCTGTGCCACACTCAGCTTTAACCCTTGT--GATGCCAACGCCCA-CTGCATCATAGAGCGGAACGGAGAGGTGTCC---TGTGCGTGCAACATTGGCTGGGCCGGTAACGGGAACACTTGTGGCACAGACACAGACATTGATGGATACCCAGACCGCTCTCTGCCCT
TSP3B    TCCTGCGGAGGTTCTGCGCCACGCTGGGCTTCAACCCCTGT--GACGTCAACGCCCA-CTGTGTCATAAAGGGTGCTGCCAGGGTCTCC---TGCGAGTGTAATGTGGGCTGGGCAGGTAATGGGCATACATGTGGCCCAGACACAGACATTGATGGCTACCCCGACCAACCTCTCCCCT
TSP4B    GTGGTGGGAGGCTCTGTGGGAATGGCCAGCCCAACCCCTGT--GACAGCAATGCACA-GTGTGTAGTTGAGAGAGACGGGAGTGTTAGC---TGCGAGTGTGACATAGGGTGGGCAGGTAATGGCTATGTGTGTGGGAAGGACACAGACATCGATGCTTACCCAGACGACAAGCTAAGGT
TSP1     CACCACGTAACCCCTGCCTGGATGGGAGCCACGAATGCAACAAGAATGCCCGCTGTAACTACCTGGGACAATTTGCCGACCCCATGTACCGCTGTGAGTGTAAGCCTGGCTATGCTGGAAATGGACACATCTGTGGTGAGGACACGGACCTGGACGGATGGCCCAACCAAGACCTGGTCT
TSP2     AGCCAGAGAACCCCTGCAAAGACAAGACCCACAACTGTCACCGGTCCTCAGAGTGTATCTACATCAGCCACTTTAGTGAACCCATGTACAAGTGTGAGTGCAGGATCGGCTACGCTGGTGATGGCTTCATCTGTGGAGAGGACTCAGACCTGGATGGATGGCCTAATCAGAACTTGGTGT
                     *     ***                 *         *     *        *  *    *      *        *       *    *    ** * ***         ** *  ** **  * **         *****     *** * *** * ** *  *  **  *          *    *

TSP3A    GTATGGACAATGAC---AAGCACTGTAAACAGGACAACTGTGTTTACACACCCAACTCAGGCCAGGAGGACGCAGACAACGACGGCATCGGAGACCAGTGTGACGAGGACGCAGATGGGGACGGCATCAAGAATGTGGAGGATAACTGTCGACTAGAACCCAACAAAGATCAGCAGAACT
TSP3B    GCATAGACAATGAC---TATCACTGCCGAGCCGACAACTGTGTGAACACACCCAACTCTGGCCAGGAGGATGCAGACGGAGACGGTATAGGTGACCAGTGTGATGAAGATGCGGACGGAGACGGCATCAAGAACGTGGAGGACAACTGCCGTCTGGTCCCAAACAAGGATCAACAGAACT
TSP4B    GCAGGGACAACA---------ACTGTAAGAAGGACAACTGTATGTTTGTTCCTAACTCTGGCCAAGAGGACGCAGACAGGGACGGGCTCGGGGACGCCTGTGATGATGACGCTGATAGCGACGGCATCGTTAACATAGAGGATAACTGTTGGCTGCATCCTAACGTGAACCAGAAGAACA
TSP1     GTGTGGAGAACGCCACCTACCACTGCAAGAAGGACAACTGCCCCAACCTTCCCAACTCAGGCCAAGAAGACTACGACAAGGATGGTGTTGGTGACGCTTGTGACAACGATGATGACAATGATGGTATTGCTGACGACAGGGATAACTGTCCGTTCGTGTACAACCCCAGGCAATATGACT
TSP2     GTGGGGCCAACGCCACCTATCACTGCAAAAAGGATAACTGCCCCAACCTGCCTAACTCTGGACAGGAAGATTTTGACAAAGATGGTCAGGGAGACGCGTGTGACCAGGATGATGACAACGATGGAATCGTGGATGAAAGAGACAACTGTCCTCTAATGTACAATCCCAGACAGTTTGACT
         *    *  **            ****       ** *****           ** ***** ** ** ** **     ***    ** **     ** ***    *****  * ** *  **     ** ** **      *        ** *****      *        **        **      **

TSP3A    CAGACACAGACTCTTTCGGTGACGCCCGTGACAACTGTCCCAATGTCCCTAACATTGACCAGAGGGATACGGACAGCAACGGGCAAGGAGACGCCTGTGACAACGACATAGATGGAGATGGTATCCCCAACGTGCTAGACAACTGCCCAAAGTCCCCAACCCCATGCAGACAGACAGG
TSP3B    CGGACACAGACTCGTATGGGGACACGTGTGACAACTGCCCCAACGTTCCCAACGGAAGCCAGAAGGACACAGATGGAAACGGGGCTGGGGATACCTGTGACAATGACATTGATGGGGATGGTATCCCAAACGTGCTGGACAACTGTCCCAAGGTGCCCAATCCTATGCAGACTGACCGTG
TSP4B    GCGATAAGGATCTCCATGGCGACGCGTGTGACAACTGCTTGACGACGGAGAACCCTGATCAACGAGACACGGACAAGGACGGGCTAGGCGATGACTGTGACGATGACATGGACGGAGACGGCCTGAAGAACATTCTGGATAACTGCCAGCGTGTGGCCAACCTAGACCAGAGGGACAGAG
TSP1     ATGACAGAGATGATATTGGAGATCGTTGTGATAACTGTCCTTACAACAGCAACCCTGACCAGACCGACACAGACAACAACGGTGAAGGAGACGCCTGCTCTGTGGACATTGATGGAGATGGCATTCTGAATGAGAAAGACAACTGCCCGTACGTCTACAACGTGGACCAGAAAGACACTG
TSP2     TTGATAAGGATGATGTGGGGGATCGTTGTGACAACTGCCCCTATGAACACAACCCTGCCCAGATCGACACCGACCACAATGGGGAAGGTGACGCCTGTGCTATCGACATTGATGGAGATGAAATCCTAAACGAGCAAGACAACTGCCCGTACTTGTACAACAATGACCAGAAGGACACTG
           ** *  **        ** **      **** *****               ***       **      ** ** **      * **      ** **    ***          ***** ** ** ** *    *      **          ** ***** *        *    ***          ****  ***     *

TSP3A    ATGGAGACGGGGTAGGAGACGCCTGCGACAGCTGCCCTGAGATCAGCAACCCCATGCAGACGGACATTGACAATGACCTGGTGGGGGATGTGTGTGACACTGACCAGGACACGGATGGGGACGGTCACCAGGATTCCAGGGACAACTGCCCTGACCATCCCAACAGCTCCCAGCTGGACT
TSP3B    ATGGGGATCGAGTGGGGGACGCTTGTGACAGCTGCCCGGAAGTCAACGACCCCATGCAGTCTGATACAGACAACGACCTGGTGGGAGATGTGTGTGACACCAACCAGGACATGGATGGGGACGGCCACCAGGATTCAAGGGACAACTGTCCAGACGTCCCTAACAGCTCCCAGCTGGACT
TSP4B    ACAACGATGGGGTGGGAGACGCCTGTGACAGCTGTCCTGACATGGTCAACCCCAACCAGTCAGATGTTGATGATGATCTTGTAGGAGACACATGTGACACCAACATAGACAGTGATGGGGACGGTCACCAGAACACTAAGGACAACTGTCCTACAGTCATCAACTCCTCCCAGCTGGACA
TSP1     ACCTGGACGGGGTGGGAGACATGTGTGACAACTGTCCTCTGGAACACAACCCTGACCAGGTTGACTCTGATGACGATCGTGTGGGAGATAAGTGTGACAGCAACCAGGACACTGATGAAGATGGCCACCAGAACAACCTGGACAACTGCCCCTACATCCCCAACGCTAACCAGGCCGACC
TSP2     ACATGGATGGAGTCGGTGACCAGTGTGACAACTGTCCTCTGCTACACAACCCTGACCAGGCTGACACAGACAATGACCTTGTAGGAGACCAGTGTGACAACAACCAGGATATCGATGAAGACGGTCACCAGAATAACCTGGACAACTGTCCTTACGTCGCCAACTCCAACCAGGCCGACC
         *    **  * ** ** ***   ** **** *** **          * ****    ***    **     **  * ** *  ** ** **     *******    **    ** *  ****  ** ** ****** *       ********* **                ***      ****    ***

TSP3A    CGGACAATGATGGCCTTGGGGACGATTGTGACGATGACGATGACAATGACGGATTCCCAGACGTA------------CAGGACAACTGCAGACTTATCACCAATCCCAACCAGAAAGATTCTAACAGTAACGGGGTGGGCGATGTGTGTGAGAACGACTTTGACAACGATTCAGTCTGGG
TSP3B    CAGACAATGACGGCATCGGCAACGAATGCGACGATGACGACGACAACGATGGCATTCCAGATGCGAGACCTCCCGGGCCGGATAACTGTCGTCTCATCCCTAACCCCAGCCAGACAGACACTAATGGAAATGGCATTGGTGACATGTGCGAGAATGACTTTGACAACGACTCCGTGATAG
TSP4B    CAGATAAGGACGGACAGGGAGACGAGTGTGACGATGACGATGACAACGACAGTATCCTGGACCAA------------GCAGACAACTGCAGACTGGTGGTCAACCCTGACCAGACAGATGAGGACAATGACGGCGTGGGTGATGCGTGTGCAGGGGACTTTGACCAGGACAAGGTGATTG
TSP1     ACGACAAAGACGGCAAGGGAGACGCCTGCGACCACGATGATGACAATGACGGGATCCCAGACGAG------------AAAGATAACTGCAGACTGGCCTTCAACCCTGACCAGATCGACTCCGATGGTGATGGTCGAGGAGACGCTTGCAAAGATGACTTTGACCAAGACAACGTCCCTG
TSP2     ACGATAAGGACGGCAAGGGGGACGCCTGTGACTTCGACGATGACAATGACGGTATCCCTGACGAT------------AGGGACAACTGTAGATTGACACCCAATAAAGATCAACTGGACTCTGATGGCGACGGTAGAGGAGACGCCTGCAAAGACGACTTTGACAACGACAACATCCCAG
            ** ** ** **     **  ***   ** ***    ** ** ***** **   *   * *   **                      ** *****  *   *        **        **      **       *       * **       ** **       **             ********* * **        *     *
```

```
TSP3A   ATCTGATTGATGTGTGCCCTGAGAGTTCAGAGGTCACACTGACAGACTTCAGAGCCTATCAGACAGTCATTCTGGATCCAGAGGGCGATGCCCAGATCGATCCCAATTGGGTGGTGCTCAATCAGGGCATGGAAATAGTTCAGACCATGAACAGTGATCCTGGTTTAGCTGTGGGCTACA
TSP3B   ACTTGATTGACGTGTGTCCAGAAAGCGCTGAAGTCACAATGACCGACTTCAGAAACTATCAGACGGTCATCCTGGACCCGGAGGGCGATGCCCAAATCGAACCCAAGTGGATTGTGCTCAATCGGGGTATGGAAATTGTTCAAACCATGAACAGTGACCCTGGTTTGGCCATAGGCTACA
TSP4B   ACAGGATAGACAACTGTCCAGAGAATGCTGAGGTCACCCTGACAGACTTCAGAGCCTATCAGACGGTAGTACTGGACCCTGAGGGTGACGCCCAGATTGATCCCAACTGGGTGGTTCTCAACCAGGGTATGGAGATAGTGCAGACTATGAACTCTGACCCTGGCCTCGCTGTAGGCTACA
TSP1    ACATCTACGACGTGTGTCCGGAGAACTTTGACATCAGCGAGACGGACTTCAGGAAGTTCCAGATGGTTCCTCTGGACCCCAAGGGAACGTCCCAGATCGACCCCAACTGGGTGGTCAGACATCAGGGCAAAGAGCTGGTCCAGACCGTCAACTGTGACCCTGGCATTGCTGTTGGTTTTG
TSP2    ACTTCCTTGACGTGTGTCCTGAGAACAATGCCGTCAGCGTCACAAACTTCCGGAAGTTTCAGATGGTGCACCTGGACCCCAAGGGAACAGCGCAGATCGACCCCAACTGGGTGGTTAGACATCAGGGCAAGGAGCTGGTACAGACCGTCAACTCTGACCCAGGCATCGCTGTTGGCTTTG
        *       **    ** ** ** *      *   ***      **  ***** *    *  ****  **     ***** **  ****      * ** ** ** ***** *** * **      * * *** *  **  * ** ** **  * ***  *** ** **  * **  * ** *

TSP3A   CAGCGTTCAACGGGGTGGACTTTGAGGGCACCTTCCACATCAACACGGTGACGGACGACGACTATGCAGGCTTCATCTTCGGCTACCAGGACTCCTCTTCCTTCTACGTGGTGATGTGGAAACAGACAGAGCAGACCTACTGGCAGTCGACACCCTTCAGGGCCATGGCCCAACCTGGCC
TSP3B   CAGCCTTTAACGGGGTAGACTTTGAAGGGACATTCCACATCAACACGGCCACAGACGATGACTACGTGGGCTTCATCTTCGGTTACCAGGACTCGTCCAGCTTCTACGTGGTCATGTGGAAACAGACGGAGCAGACCTACTGGCAGTCGCTGCCTTTTAGGGCCAGGGCCCAGCCAGGCC
TSP4B   CAGCGTTCAGTGGGGTGGACTTTGAGGGCACGTTCCACGTGAACACAGTGACTGATGATGACTATGCAGGCTTCATCTTCGGCTACCAGGACTCCTCCTCCTTCTACGTGGTCATGTGGAAACAGACTGAGCAGACCTACTGGCAGGCTGTACCCTTCAGAGCTGTGGCTGAGCCTGGCA
TSP1    ATGAGTTCAACTCTGTGGACTTCAGTGGTACCTTCTTCATCAACACAGAGCGAGATGATGACTACGCCGGCTTTGTGTTCGGCTACCAGTCCAGCTCCAGGTTCTACGTGGTGATGTGGAAGCAGATCACACAGACCTACTGGAACAACAAACCCACCAAGGCTCAGGGATACTCAGGAC
TSP2    ATGAGTTCAACGCGGTGGACTTCAGTGGTACGTTCTACGTGAACACGGACCGGGATGACGACTATGCTGGCTTTGTGTTCGGTTACCAGTCGAGCGGCCGCTTCTACGTGGTGATGTGGAAGCAGATCACTCAGACCTACTGGGAAGACAAACCCTCCAAGGCCTTTGGTATCTCTGGTG
          *  ** *     ** *****    ** ** ***  * * ***** *     ** ** ***** *  *****  * ***** ******            *********** ******** ****      *********** *       **    *  **    *      * **

TSP3A   TGCAGCTCAAAGCAGTGAAGTCCCGTACAGGGCCTGGTGAGTACCTGCGTAACGCCCTGTGGCACACAGGGGACACCAACGAGGAGGTGAAGCTGCTGTGGTCGGACCCACGGAACGTTGGCTGGAGAGACAAGACGTCTTACCGCTGGCAGCTCAGCCACAGGCCCCAGGTGGGCTACA
TSP3B   TGCAACTAAAGGCAGTGAAGTCACGTACGGGTCCAGGCGAGTACCTCCGGAACGCCCTGTGGCACACGGGCGACACCCCGGGCGAGGTCACCCTGCTGTGGAAGGACCCCCGGAATGTGGGCTGGAGGGACAAGACATCTTACCGCTGGCACCTCAGCCACCGCCCACAGATGGGCTACA
TSP4B   TACAGCTCAAGGCAGTGAAGTCTAAGACAGGTCCAGGGGAGTATCTGAGGAACTCCCTGTGGCATACAGGAGACACCAAGGACCAGGTACGTCTGCTGTGGAAGGACAAGAGGAACGTGGGTTGGAAGGACAAGGTGTCCTACCGCTGGTATCTACAGCACAGACCACAGGTTGGATACA
TSP1    TGTCCATCAAGGTGGTCAACTCCACTACTGGACCTGGAGAGCACCTCAGGAACGCTCTCTGGCACACTGGTAACACCCCAGGACAGGTGAAGACTCTTTGGCACGACCCTAAGAACATTGGCTGGAAAGACTTCACAGCCTACAGATGGCACCTGATCCACAGACCAAGAACAGGACACA
TSP2    TCTCTCTCAAGGTGGTCAACTCAACTACGGGCACAGGGGAGAACCTGAGAAATGCCCTGTGGCACACAGGCAACACAAAAAATCAGGTGCGCACTCTGTGGCACGACCCAAAGAACATTGGCTGGAAGGATTACACTGCCTACAGGTGGCACCTCATCCACAGACCCAAGACTGGATTCA
        *     * ** *  ** ** **    ** **  * ** *** * **  * **  * ** ***** ** **  ****         ****       ** ***    ***      ***  * ** ****  **        * *** * *** * **    *** * **        **   **

TSP3A   TCAGGGTGAAGTTATATGAGGGGACGGAGATGGTGGCTGACTCTGGCGTGGTGATTGACACTACCATGAGAGGCGGACGACTGGGGGTCTTCTGTTTCTCCCAAGAGAACGTCATCTGGTCCAACCTGCGCTACCGCTGCAATGACACTGTTCCAGACGACTTCAACCCATATCGCAAAC
TSP3B   TCAAAGTGAGGCTGTATGAAGGCATGACATTGGTGGCAGACTCGGGCGTGCTGGTGGACACCTCAATGAGGGGCGGGAGGCTGGGCGTCTTCTGCTTCTCCCAGGAGCAAATCATCTGGTCCAACCTGGGCTATCGCTGCAACGATACTGTACCAGAGGACTATGAGTTCTACCGCAAGC
TSP4B   TCAGGGCGCGTTTCTATGAGGGTCCCAACCTGGTAGCAGATTCTGGGGTGAAGATAGACAACAGTATGCGAGGAGGGAGACTGGGAGTCTTCTGTTTCTCCCAGGAAAACATCATCTGGTCCAACCTCAAGTACCGCTGCAATGACACCATCCCTGAAGATTACCAGGAGTACAGTGCCC
TSP1    TCAGAGTGGTCATGTACGAGGGGAAGAAGATTATGGCTGATTCTGGGAGCATATATGACAAGACGTACGCAGGCGGAAGACTAGGCCTCTTTGTCTTCTCGCAAGAGATGGTATACTTCTCAGACCTCAAGTATGAATGCAGAGATGCATAA---------------------------
TSP2    TTAGGGTTGTGGTCTATGAAGGGAAACAGATCTTGGCAGACTCGGGACCAGTGTACGACAAGACGTTTGCTGGAGGACGGTTAGGCCTGTTTGTCTTCTCCCAGGAACTGGTCTTCTTCTCTGATCTCAAATATGAGTGTAGAGATAACTGA---------------------------
        * *  *       * ** ** **         *  * ** ** ** **           ****            ** **  *  * **  * **    ***** ** **     *   **  **  * **    **    ** *  **

                                        ORF length (bp)
TSP3A   AGGTTCTGCTGCACATCAAGGTGTGA      2868
TSP3B   AGATCC------ACATCAGGGTGTGA      2868
TSP4B   AGAACAC----TGAATAA--------      2844
TSP1    --------------------------      3519
TSP2    --------------------------      2961
```
