# Supplementary figures and images for: Dataset for characterization of thrombospondin family in chum salmon (Oncorhynchus keta)
Source: Data Brief. 2019 Jan 9;22:866–70. doi: 10.1016/j.dib.2019.01.008 (PMC6362859; doi:10.1016/j.dib.2019.01.008)

(A)

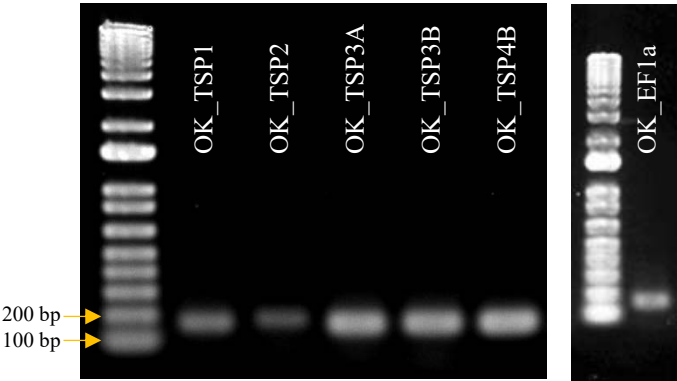

(B)

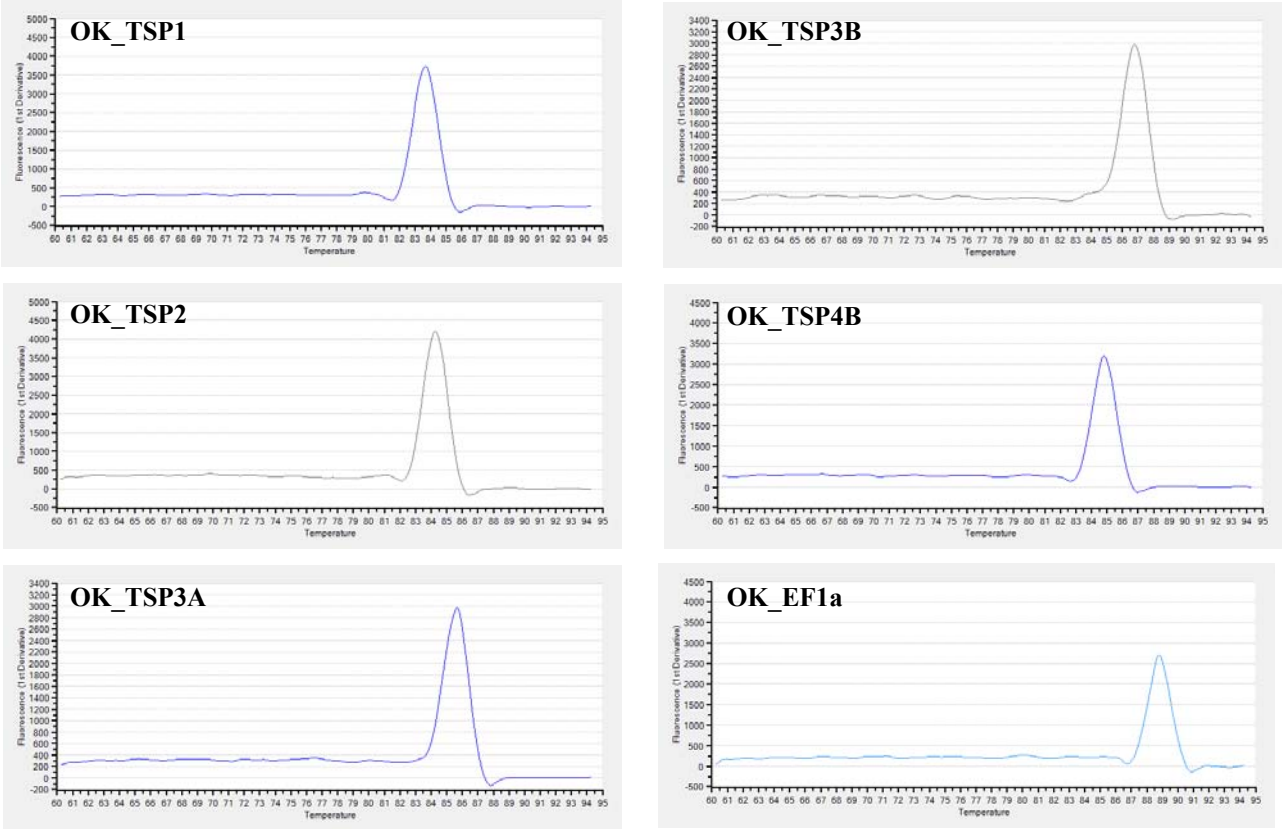

Supplement: Supplementary file 4 — Supplementary material Supplementary C. Agarose gel electrophoresis of RT-PCR products show single amplicons (A) and melt curves from qPCR of chum salmon TSP isoform and EF1a genes (B). [file mmc4.pdf]
